# FGF19‐Induced Inflammatory CAF Promoted Neutrophil Extracellular Trap Formation in the Liver Metastasis of Colorectal Cancer

**DOI:** 10.1002/advs.202302613

**Published:** 2023-06-22

**Authors:** Chen Li, Tianli Chen, Jialiang Liu, Yue Wang, Chunhuan Zhang, Lu Guo, Dandan Shi, Tingguo Zhang, Xishan Wang, Jie Li

**Affiliations:** ^1^ Department of Ultrasound Qilu Hospital of Shandong University Jinan Shandong 250012 China; ^2^ Department of Colorectal Surgery National Cancer Center/National Clinical Research Center for Cancer/Cancer Hospital Chinese Academy of Medical Sciences and Peking Union Medical College Beijing 100021 China; ^3^ Department of General Surgery Qilu Hospital of Shandong University Jinan Shandong 250012 China; ^4^ Department of Clinical Laboratory Qilu Hospital of Shandong University Jinan Shandong 250012 China; ^5^ Department of Pathology Qilu Hospital of Shandong University Jinan Shandong 250012 China

**Keywords:** FGF19, fisogatinib, inflammatory cancer‐associated fibroblast, liver metastasis, neutrophil extracellular trap

## Abstract

Liver metastasis is the main cause of death in patients with colorectal cancer (CRC); thus, necessitating effective biomarkers and therapeutic targets for colorectal cancer liver metastasis (CRLM). Fibroblast growth factor 19 (FGF19) is a protumorigenic gene in numerous human malignancies. In this study, it is shown that FGF19 plays an indispensable role in CRLM. FGF19 expression and secretion are markedly correlated with liver metastasis and lower overall survival rates of patients with CRC. An in vivo metastasis model shows that FGF19 overexpression confers stronger liver‐metastatic potential in CRC cells. Mechanistically, FGF19 exerts an immunomodulatory function that creates an environment conducive for metastasis in CRLM. FGF19 mediates the polarization of hepatic stellate cells to inflammatory cancer‐associated fibroblasts (iCAFs) by activating the autocrine effect of IL‐1*α* via the FGFR4‐JAK2‐STAT3 pathway. FGF19‐induced iCAFs promote neutrophil infiltration and mediate neutrophil extracellular trap (NET) formation in liver metastatic niches via the production of complement C5a and IL‐1*β*, which in turn accelerates the liver colonization of CRC cells. Importantly, targeting FGF19 signaling with fisogatinib efficiently suppresses FGF19‐induced liver metastasis in a mouse model. In summary, this study describes the mechanism by which FGF19 regulates CRLM, thereby providing a novel target for CRLM intervention.

## Graphical abstract

Liver metastasis is the main cause of colorectal cancer (CRC)‐related deaths in humans. Tumor‐secreted FGF19 mediates the polarization of hepatic stellate cells to inflammatory cancer‐associated fibroblasts (iCAFs), thereby modulating neutrophil infiltration and the metastasis‐supporting neutrophil extracellular traps (NETs) in liver metastatic niches. These promote liver colonization of CRC cells and support FGF19‐targeting therapy for colorectal cancer liver metastasis.

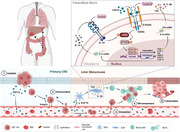

## 1 Introduction

Colorectal cancer (CRC) is the third most common cancer and the second leading cause of cancer‐related deaths worldwide.^[^ ^1^ ^]^ The liver is the primary organ for CRC metastasis, and liver metastasis is the predominant cause of CRC‐related deaths.^[^ ^2^ ^]^ Despite the advancement of surgical techniques and systemic therapies in recent years, the prognosis of patients with colorectal cancer liver metastasis (CRLM) remains unsatisfactory, with a 5‐year survival rate of less than 15%, which is the main challenge for current clinical treatment.^[^ ^3^, ^4^ ^]^ Hepatic resection is the only option for the long‐term survival of patients with CRLM; however, more than 80% of patients would have lost the window of opportunity for surgery at the time of diagnosis.^[^ ^2^, ^5^ ^]^ Therefore, new biomarkers and targeted therapies are urgently needed to improve the survival of patients with CRLM.

The tumor microenvironment (TME), consisting of various cell types and extracellular matrix components, is essential in tumor metastasis.^[^ ^6^ ^]^ Cancer‐associated fibroblasts (CAFs) are protumorigenic components of the TME, as they participate in desmoplasia, immunosuppression, and secretion of factors that promote seeding and outgrowth of tumor cells.^[^ ^7^, ^8^ ^]^ According to recent single‐cell sequencing analyses, CAFs are a heterogeneous cell population that include inflammatory CAFs (iCAFs) and myoblastic CAFs (myCAFs).^[^ ^8^, ^9^, ^10^ ^]^ iCAFs, characterized by IL‐1*α*‐ and LIF‐dependent cytokine cascades, contribute to tumor progression and therapy resistance through the secretion of immunomodulatory and protumorigenic factors.^[^ ^8^, ^11^, ^12^ ^]^ Meanwhile, the functional role of myCAFs in tumor growth and metastasis remains controversial, as some studies report that myCAF‐produced type I collagen restricts, rather than promotes, tumor progression.^[^ ^9^, ^13^ ^]^ These studies indicate distinct functions of different CAF subtypes in the TME, and additional studies are required to elucidate the interregulation between tumor cells and CAFs.

Furthermore, neutrophils account for a significant portion of cells in the TME in several malignancies. Accumulating evidence has shown the promoting effect of neutrophils on tumor proliferation, metastasis, and immune escape.^[^ ^14^, ^15^, ^16^ ^]^ Neutrophil extracellular traps (NETs), which employ a form of neutrophil death distinct from apoptosis and necrosis, were recently demonstrated to remodel the TME to attract and capture tumor cells and drive their progression.^[^ ^17^, ^18^, ^19^, ^20^ ^]^ Well‐founded evidence supports that highly metastatic tumor cells can induce NETosis, which is the NET‐forming process of neutrophils, in target organs, such as the liver, lung, and omentum, to facilitate tumor cell colonization.^[^ ^18^, ^21^, ^22^, ^23^ ^]^ Tumor‐secreted soluble factors, such as IL‐8 and IL‐17, induce NETosis.^[^ ^20^, ^24^ ^]^ In addition, CAFs induce NET formation, driven by a reactive oxygen species‐mediated pathway dependent on CAF‐derived amyloid *β*.^[^ ^25^ ^]^

A critical and rate‐limiting step for tumor metastasis is the colonization of tumor cells in target organs.^[^ ^26^ ^]^ According to the “seed and soil” theory of Paget,^[^ ^27^ ^]^ tumor cells (seed) can cultivate a metastasis‐supporting microenvironment (soil) in target organs prior to their arrival, which is conducive to the early‐stage seeding and outgrowth of tumor cells. This abnormal and metastasis‐supporting microenvironment is defined as the premetastatic niche (PMN).^[^ ^28^ ^]^ Orthotopic tumor‐derived soluble molecules, such as growth factors, inflammatory cytokines, chemokines, and exosomes, are pivotal for the formation and regulation of the PMN. Therefore, the identification of the secreted factors involved in the formation of metastasis‐supporting “soil” will provide novel insights into the biomarkers and therapeutic targets for the diagnosis and treatment of malignancies.^[^ ^29^ ^]^

Fibroblast growth factors (FGFs) are oncogenes for various malignancies.^[^ ^30^ ^]^ In recent years, targeted therapies for the FGF‐FGF receptor (FGFR) have emerged gradually. In addition to US FDA‐approved erdafitinib (bladder cancer) and pemigatinib (cholangiocarcinoma), targeted drugs infigratinib and rogaratinib have also undergone phase II/III clinical trials and achieved satisfactory effects.^[^ ^31^ ^]^ FGF19 is a member of the endocrine FGF subfamily. FGF19 plays crucial roles in various physiological processes, such as glucose, lipid, and bile acid metabolism.^[^ ^32^ ^]^ Pathologically, FGF19 mediates the progression of malignant tumors, including CRC, hepatocellular carcinoma (HCC), and gallbladder carcinoma (GBC), by activating the FGFR4/*β*‐Klotho complex and downstream signals.^[^ ^33^, ^34^, ^35^, ^36^ ^]^ Current studies on FGF19 mainly focus on its effects on tumor cell intrinsic malignancy; however, little is known about its regulation of nonmalignant cells in the TME.

In this study, we examine the clinical relevance of FGF19 in the liver metastasis of human CRC. We found that CRC cell‐derived FGF19 promotes the formation of iCAFs and subsequent NETs in liver metastatic niches to facilitate CRLM. In addition, fisogatinib, which is an inhibitor targeting the FGF19‐FGFR4 complex, has been assessed in a phase I trial of patients with advanced HCC. For patients with FGF19‐ positive HCC, fisogatinib treatment was well tolerated and was a good cancer intervention.^[^ ^37^ ^]^ Our study confirmed that FGF19 blockade with fisogatinib efficiently suppresses the liver metastasis induced by FGF19‐overexpressing CRC cells in a mouse model. Taken together, our findings suggest FGF19 as a novel prognostic marker and therapeutic target for CRLM.

## 2 Results

### 2.1 FGF19 Was Associated with CRLM

**Figure 1:**
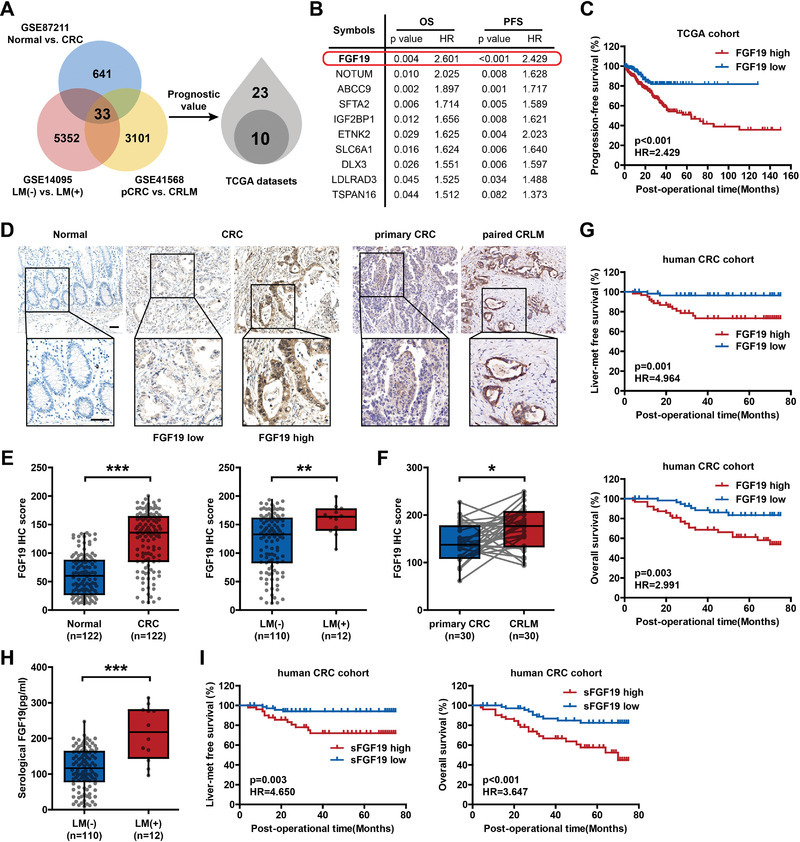
High FGF19 expression is correlated with liver metastasis in colorectal cancer. A) Flowchart of CRLM‐related gene screening using the bioinformatics method. Left panel: Venn diagram showing the 33 overlapping DEGs (log_2_ |fold change|≥2 and *p*‐value < 0.05) in three CRC‐ and CRLM‐related datasets from the Human Genome Array. Right panel: The prognostic value of the 33 overlapping genes was further validated in TCGA CRC cohort. Ten candidates were screened out. B) Among the 10 candidates, FGF19 was confirmed to have the strongest prognostic value. C) Kaplan‒Meier survival curves of progression‐free survival for CRC patients with low (*n* = 247) and high (*n* = 161) FGF19 expression. Patients were stratified into FGF19‐high or FGF19‐low groups according to the cutoff value. D) Representative IHC staining of FGF19 expression in normal colon epithelium, CRC, and paired primary CRC and CRLM tissues. Scale bar: 50 µm. E) FGF19 expression was higher in CRC tissues (*n* = 122) than that in normal colon epithelium tissues (*n* = 122), and higher in LM(+) CRC tissues (*n* = 12) than that in LM(−) CRC tissues (*n* = 110). F) FGF19 expression was higher in CRLM tissues (*n* = 30) than that in paired primary CRC tissues (*n* = 30). G) Kaplan‒Meier survival curves of overall and liver metastasis‐free survival of CRC patients with low (*n* = 59) and high (*n* = 63) FGF19 expression. H) The serological level of FGF19 was higher in patients with LM(+) CRC (*n* = 12) than that in those with LM(−) CRC (*n* = 110). I) Kaplan‒Meier survival curves of overall and liver metastasis‐free survival of CRC patients with low (*n* = 71) and high (*n* = 51) serological levels of FGF19. *p < 0.05, **p < 0.01, ***p < 0.001. In (E) and (H), data were subjected to Mann–Whitney test (data not normally distributed). In (F), data were subjected to paired Student's *t*‐test. In (B), (C), (G), and (I), data were subjected to Cox proportional hazards regression. See also related Figure S1, Supporting Information.

To investigate CRLM‐associated genes, we first screened three differentially expressed gene (DEG) datasets, including 641 genes upregulated in CRC tissues compared with normal tissues (GSE87211), 5352 genes upregulated in CRC tissues with liver metastasis (LM(+)) compared with those without (LM(−)) (GSE14095), and 3101 genes upregulated in CRLM tissues compared with primary CRC (pCRC) tissues (GSE41568). Overlapping analysis identified 33 DEGs that were consistently upregulated in all of these datasets. Using The Cancer Genome Atlas (TCGA) database, we further performed survival analysis on these 33 candidates, 10 of which were correlated with poor prognosis of patients with CRC (**Figure** **1**A). Comparison of the hazard ratio of these genes (Figure 1B) revealed that FGF19 was closely associated with CRLM and had the strongest prognostic value (Figure 1C; Figure S1A,B, Supporting Information).

To further validate the clinical relevance of FGF19 expression, we analyzed a cohort of 122 human CRC tissues via immunohistochemical (IHC) staining of FGF19 and found that FGF19 expression was higher in CRC than that in normal tissues and higher in LM(+) CRC than that in LM(−) CRC tissues, consistent with previous results (Figure 1D,E). Meanwhile, we detected FGF19 expression in 30 primary CRC and paired liver metastasis tissues and confirmed FGF19 upregulation in CRLM (Figure 1D,F). Its coreceptor *β*‐klotho was also expressed at higher levels in metastatic tumors (Figure S1C, Supporting Information). Patients with higher FGF19 expression in primary tumors were prone to synchronous and subsequent liver metastasis and shortened overall survival (OS, Figure 1G; Figure S1D,E, Supporting Information). FGF19 serves as an endocrine hormone;^[^ ^32^ ^]^ thus, an enzyme‐linked immunosorbent assay (ELISA) was used to measure FGF19 levels in the serum of patients with CRC (Figure S1F, Supporting Information). Serological FGF19 (sFGF19) was significantly upregulated in patients with CRLM than that in patients without liver metastasis (Figure 1H). More importantly, a higher level of sFGF19 was markedly associated with metastasis to the liver and lower OS of patients with CRC (Figure 1I; Figure S1G,H, Supporting Information). Collectively, these results strongly support the notion that FGF19 is a reliable biomarker for liver metastasis and poor prognosis in patients with CRC.

### 2.2 FGF19 Promoted Liver Metastasis in CRC

**Figure 2:**
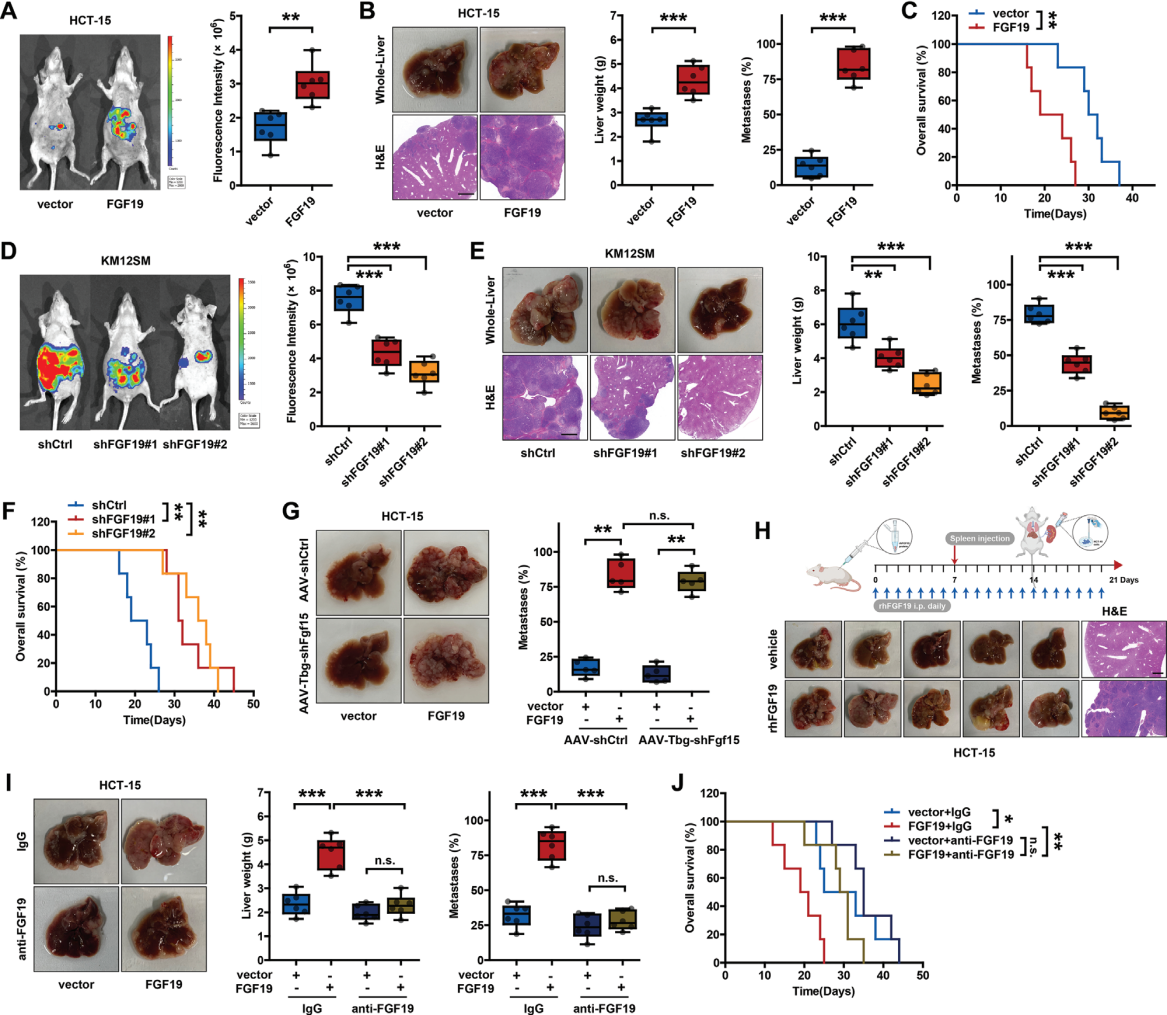
FGF19 promoted CRLM in a mouse model. A–C) Splenic injection of HCT‐15 cells with FGF19 overexpression for liver metastasis experiments (*n* = 6 mice). A) IVIS images and quantification, B) liver weight and the proportion of tumor metastases in livers, and C) animal overall survival are shown. Scale bar: 1 mm. D–F) Splenic injection of KM12SM cells with FGF19 knockdown for liver metastasis experiments (*n* = 6 mice). D) IVIS images and quantification, E) liver weight and the proportion of tumor metastases in livers, and F) animal overall survival are shown. Scale bar: 1 mm. G) Mice were administrated intravenously with AAV‐shCtrl or AAV‐Tbg‐shFgf15 (1.0 × 10^12^ genome copies per mouse) for 4 weeks and then injected FGF19‐overexpressing HCT‐15 cells for liver metastasis experiments (*n* = 5 mice). The proportion of tumor metastases in livers is shown. H) Experimental design of rhFGF19 (50 µg kg^−1^) treatment and sequential splenic injection of HCT‐15 cells in a liver metastasis mouse model. Liver specimens (*n* = 5 mice) were harvested 14 days after splenic injection of HCT‐15 cells. Scale bar: 1 mm. I,J) Liver metastasis of mice with splenic injection of FGF19‐overexpressing HCT‐15 cells treated with or without FGF19 neutralizing antibody (25 µg per mouse) (*n* = 6 mice). I) Liver weight and the proportion of tumor metastases in livers and J) animal overall survival are shown. *p < 0.05, **p < 0.01, ***p < 0.001; and n.s., nonsignificant. In (A), (B), (D), (E), (G), and (I), data were subjected to Student's *t*‐test (data normally distributed). In (C), (F), and (J), data were subjected to log‐rank test. See also related Figure S2, Supporting Information.

Next, we explored the functional role of FGF19 in CRLM. FGF19 expression was detected in different human CRC cell lines; it was detected in all cells and was expressed at a higher level in highly liver‐metastatic KM12SM cells (Figure S2A, Supporting Information). We established an in vivo CRLM model by injecting luciferase‐labeled human CRC cells into the spleens of BALB/c nude mice, and an in vivo imaging system (IVIS) was used to assess the liver metastatic burden. FGF19 overexpression in HCT‐15 and HT‐29 cells (Figure S2B, Supporting Information), two cell lines with lower endogenous FGF19 levels, significantly exacerbated the liver metastatic burden (**Figure** **2**A,B; Figure S2C, Supporting Information) and shortened animal survival (Figure 2C; Figure S2D, Supporting Information). In contrast, FGF19 knockdown via short hairpin RNA (shRNA) in KM12SM cells (Figure S2E, Supporting Information) suppressed liver colonization (Figure 2D,E) and prolonged animal survival (Figure 2F). Fgf15 (a murine orthologue of human FGF19) was also expressed in mouse hepatocytes; therefore, we generated adeno‐associated virus (AAV)‐Tbg promoter‐shRNA‐Fgf15 (AAV‐Tbg‐shFgf15) to knock down Fgf15 expression in mouse livers (Figure S2F,G, Supporting Information). FGF19‐overexpressing HCT‐15 cells showed a higher liver‐metastatic potential than control HCT‐15 cells in the absence of basal Fgf15 (Figure 2G; Figure S2H, Supporting Information). We then performed in vitro migration and invasion assays and found that FGF19 regulated the migration and invasion of HT‐29 and KM12SM cells, but not HCT‐15 cells (Figure S2I,J, Supporting Information). The loss of FGFR4 in HCT‐15 cells mainly prevented them from responding to the FGF19 signal (Figure S2K and Tables S1,  S2, Supporting Information). To confirm this, we knocked out FGFR4 using a single guide RNA in HT‐29 cells (Figure S2L, Supporting Information) and found that FGFR4 deletion abolished FGF19‐enhanced migration and invasion of HT‐29 cells in vitro (Figure S2M, Supporting Information). Interestingly, consistent with HCT‐15 cells (Figure 2A,B), FGFR4‐silencing HT‐29 cells also showed a stronger liver metastatic ability in a mouse model when FGF19 was upregulated (Figure S2N, Supporting Information). This discrepancy between in vitro and in vivo experiments indicated that FGF19 could promote CRLM through mechanisms other than tumor cell regulation via FGFR4.

Additionally, we treated BALB/c nude mice daily with recombinant human FGF19 (rhFGF19) proteins and injected HCT‐15 cells into the spleens of mice on Day 7 (Figure 2H). Treatment with rhFGF19 led to a significant increase in the number of liver metastatic nodules (Figure 2H; Figure S2O, Supporting Information). More importantly, we used an FGF19‐neutralizing antibody for the clearance of tumor‐secreted FGF19, which completely blocked the promoting effect of FGF19 on CRLM (Figure 2I,J). All these findings demonstrate the critical role of FGF19 in CRLM and suggest that it mediates CRLM in a microenvironment‐dependent manner.

### 2.3 FGF19 Mediated iCAF Formation in Liver Metastatic Niches

**Figure 3:**
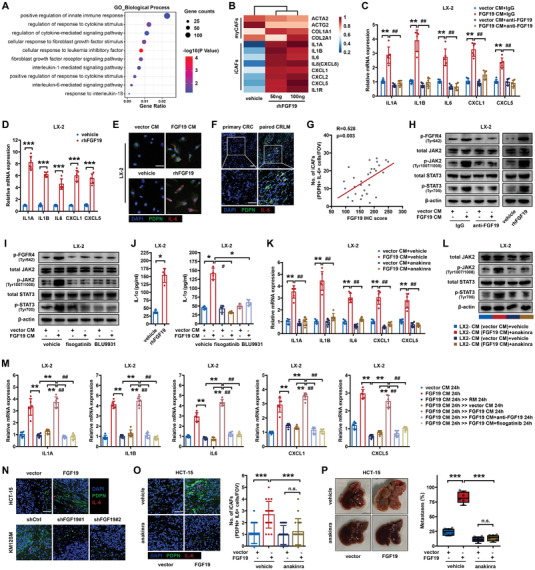
FGF19 mediated the formation of inflammatory CAFs in liver metastatic niches. A,B) mRNA sequencing of LX‐2 cells stimulated with 0, 50, or 100 ng mL^−1^ rhFGF19 for 24 h. A) Bubble plot showing the GO signatures enriched in rhFGF19‐stimulated LX‐2 cells. B) Heatmap showing the mRNA expression of iCAF and myCAF markers in rhFGF19‐stimulated LX‐2 cells. C) mRNA expression of iCAF markers in LX‐2 cells treated with FGF19‐containing CM in the presence or absence of FGF19 neutralizing antibody (10 µg mL^−1^) for 24 h (*n* = 6). D) mRNA expression of iCAF markers in LX‐2 cells treated with rhFGF19 (50 ng mL^−1^) for 24 h (*n* = 6). E) IF staining of iCAFs (PDPN+ and IL‐6+ cells) formed by LX‐2 cells treated with FGF19‐containing CM or rhFGF19 for 48 h. Scale bar: 20 µm. F) IF staining of iCAFs (PDPN+ and IL‐6+ cells) in primary CRC and CRLM tissues. Scale bar: 50 µm. G) Correlation between FGF19 expression and the frequency of iCAFs in human CRLM tissues (*n* = 30). H) Phosphorylation of FGFR4, JAK2, and STAT3 in LX‐2 cells treated with rhFGF19 or FGF19‐containing CM in the presence or absence of FGF19 neutralizing antibody for 24 h. I) Phosphorylation of FGFR4, JAK2, and STAT3 in LX‐2 cells treated with FGF19‐containing CM in the presence or absence of fisogatinib (100 nм) or BLU9931 (10 µм). J) Extracellular expression of IL‐1*α* in LX‐2 cells stimulated with rhFGF19 or FGF19‐containing CM in the presence or absence of fisogatinib or BLU9931 (*n* = 4). K) mRNA expression of iCAF markers in LX‐2 cells treated with FGF19‐containing CM in the presence or absence of anakinra (20 mg mL^−1^) for 24 h (*n* = 6). L) Phosphorylation of JAK2 and STAT3 in LX‐2 cells treated with CM obtained from LX‐2 cells pretreated with FGF19‐containing tumor cell CM, in the presence or absence of anakinra. M) mRNA expression of iCAF markers in LX‐2 cells treated with FGF19‐containing CM for 24 h and then subjected to regular medium, FGF19‐free CM, fresh FGF19‐containing CM, FGF19 neutralizing antibody, or FGFR4 inhibitor fisogatinib for 24 h (*n* = 6). N) IF staining of iCAFs (PDPN+ and IL‐6+ cells) in the livers of mice with splenic injection of FGF19‐overexpressing HCT‐15 and FGF19‐knockdown KM12SM cells (*n* = 18 RMFs from 6 mice per group). Scale bar: 50 µm. O,P) Liver metastasis of mice with splenic injection of FGF19‐overexpressing HCT‐15 cells, with or without anakinra treatment (500 mg per mouse, 250 mg morning, and 250 mg evening) (*n* = 6 mice). O) IF staining and quantification of iCAFs in the livers of mice (*n* = 18 RMFs from 6 mice per group), P) representative liver specimens and the proportion of tumor metastases in livers are shown. Scale bar: 50 µm. * or ^#^ *p* < 0.05, ** or ^##^ *p* < 0.01, *** or ^###^ *p* < 0.001; n.s., nonsignificant. In (D), (O), and (P), data were subjected to Student's *t*‐test. In (C), (J), (K), and (M), data were subjected to Mann–Whitney test. See also related Figure S3, Supporting Information.

Hepatic stellate cells (HSCs) are the main source of CAFs in liver metastases.^[^ ^9^ ^]^ FGF19 is linked to the activation of CAFs.^[^ ^38^, ^39^ ^]^ To explore the role of FGF19 in the formation and activation of CAFs, we performed mRNA sequencing (mRNA‐seq) of LX‐2 cells, a human HSC cell line, with or without rhFGF19 pretreatment (GEO ID: GSE215882; Table S3, Supporting Information). Gene ontology (GO) analysis revealed enrichment signatures of inflammation in LX‐2 cells pretreated with rhFGF19, which was consistent with the iCAF subtype identified by Biffi et al. (**Figure** **3**A).^[^ ^8^ ^]^ FGF19 increased the expression of iCAF markers, but not that of myCAF markers (Figure 3B). In addition, gene set enrichment analysis (GSEA) employing an iCAF signature gene set obtained from a recent study demonstrated a strong enrichment of iCAF‐related genes in FGF19‐treated LX‐2 cells (Figure S3A, Supporting Information).^[^ ^11^ ^]^ To further confirm the association of FGF19 with iCAFs, we treated LX‐2 cells with FGF19‐containing cancer cell conditioned medium (CM) or rhFGF19 and detected the expression of iCAF (IL1A, IL1B, IL6, CXCL1, and CXCL5) and myCAF (*α*‐SMA, ACTG2, COL1A1, COL2A1) markers. As expected, LX‐2 cells treated with FGF19‐containing CM or rhFGF19 had increased levels of iCAF markers (Figure 3C,D), but no change in the levels of myCAF markers (Figure S3B, Supporting Information). Inhibition of FGF19 with a neutralizing antibody significantly attenuated the FGF19‐induced upregulation of iCAF markers (Figure 3C). Flow cytometry analysis showed that the iCAF surface marker PDGFR*α* was overexpressed in FGF19‐conditioned LX‐2 cells, whereas the level of the myCAF marker *α*‐SMA was not altered (Figure S3C, Supporting Information). We also performed immunofluorescence (IF) staining of FGF19‐conditioned LX‐2 cells and defined iCAF phenotype based on positive staining of pan‐CAF marker podoplanin (PDPN) in combination with the well‐known iCAF marker IL‐6. FGF19 stimulation induced the polarization of LX‐2 cells toward the iCAF phenotype (Figure 3E). Moreover, in human CRLM tissues, FGF19 expression was strongly correlated with the frequency of iCAFs, as shown by IF staining (Figure 3F,G). These data suggest that FGF19 plays an important role in the polarization of HSCs to iCAFs.

To elucidate the molecular mechanism of the induction of the iCAF phenotype of LX‐2 cells by FGF19, we immunoprecipitated FGFR1‐4, EGFR, and ERBB2 and detected their phosphorylation using a pan‐phospho‐Tyr antibody after stimulating LX‐2 with FGF19‐containing CM or rhFGF19. FGFR4 tyrosine phosphorylation was significantly enhanced in FGF19‐conditioned LX‐2 cells (Figure S3D, Supporting Information). Moreover, we found that phosphorylation of FGFR4 at Y642 and downstream JAK2‐STAT3 signaling, which is a pathway required for iCAF formation, was increased by FGF19 stimulation (Figure 3H; Figure S3E, Supporting Information). Two independent FGFR4 inhibitors, fisogatinib and BLU9931, substantially attenuated FGF19‐mediated phosphorylation of FGFR4 and the JAK2‐STAT3 pathway, thereby leading to the disruption of the iCAF phenotype (Figure 3I; Figure S3F,G, Supporting Information). We also showed that the inhibition of JAK2‐STAT3 signaling reversed the inflammatory phenotype in LX‐2 cells stimulated with FGF19‐containing CM (Figure S3H, Supporting Information). These results support the FGF19‐mediated polarization of HSCs toward the iCAF phenotype via the FGFR4‐JAK2‐STAT3 axis.

Based on these findings, we examined the effect of IL‐1 signaling, which is a dominant pathway responsible for iCAF formation,^[^ ^8^ ^]^ on the induction of an inflammatory phenotype in LX‐2 cells. IL‐1*α* was upregulated in the supernatant from FGF19‐conditioned LX‐2 cells (Figure 3J; Figure S3I, Supporting Information). Treatment of LX‐2 cells with recombinant human IL‐1*α* (rhIL‐1*α*) proteins induced the activation of JAK2‐STAT3 signaling and upregulated iCAF markers (Figure S3J,K, Supporting Information). Targeting IL‐1 signaling with the IL‐1 receptor antagonist anakinra significantly reduced the induction of iCAF markers in LX‐2 cells exposed to FGF19‐containing CM (Figure 3K). Moreover, when LX‐2 cells were pretreated with FGF19‐containing tumor cell CM, the LX‐2 cells culture media could activate the JAK2‐STAT3 pathway in quiescent LX‐2 cells, whereas anakinra reversed this effect (Figure 3L; Figure S3L, Supporting Information). These results indicate that IL‐1*α* might be secreted under the influence of FGF19 and promote iCAF formation in an autocrine manner.

We also examined whether FGF19 was indispensable for the maintenance of the iCAF phenotype. We stimulated LX‐2 cells for 24 hours with FGF19‐containing CM before adding regular medium (RM), FGF19‐free CM, fresh FGF19‐containing CM, and FGF19 neutralizing antibody or the FGFR4 inhibitor fisogatinib. Replacing FGF19‐containing CM with RM or FGF19‐free CM reduced the gene expression of IL1A, IL1B, CXCL1, and CXCL5 in LX‐2 cells (Figure 3M). Neutralization of FGF19 or fisogatinib therapy also disrupted the maintenance of the iCAF phenotype (Figure 3M). These results indicate that tumor‐derived FGF19 is necessary to maintain iCAF polarization and that targeting FGF19 not only prevents but also reverses the established iCAF phenotype.

In addition, we investigated the role of iCAFs in FGF19‐mediated CRLM. IF staining confirmed the recruitment of iCAFs in the livers of mice injected with FGF19‐overexpressing CRC cells (Figure 3N). Treatment with anakinra in mice spleen‐injected with FGF19‐overexpressing HCT‐15 cells significantly suppressed FGF19‐induced iCAF formation in liver metastases (Figure 3O), thereby attenuating CRLM (Figure 3P; Figure S3M, Supporting Information). Therefore, we demonstrated that CRC‐derived FGF19 promotes iCAF formation in liver metastatic niches to regulate CRLM.

### 2.4 Neutrophils Were Involved in Liver Metastasis Mediated by FGF19

We investigated how FGF19‐induced iCAFs promote CRLM. Given that iCAFs secrete abundant cytokines and inflammatory factors, we mainly examined the changes in stromal components of various immune cell populations, including neutrophils, macrophages, natural killer (NK) cells, B cells, and dendritic (DC) cells, in liver metastases caused by FGF19‐overexpressing or ‐knockdown CRC cells. IF staining showed that FGF19 overexpression increased, whereas FGF19 knockdown reduced the percentages of Ly‐6G+ neutrophils, but not other immune cells, in liver metastases (Figure S4A, Supporting Information). Disruption of the iCAF phenotype with anakinra blocked the infiltration of Ly‐6G+ neutrophils induced by FGF19 overexpression (Figure S4A, Supporting Information). GSEA of mRNA‐seq data also revealed that FGF19‐stimulated LX‐2 cells might affect neutrophil migration and chemotaxis (Figure S4B, Supporting Information). Through transwell assays, we confirmed that FGF19‐conditioned LX‐2 cells regulated neutrophil migration in vitro, and this effect was inhibited by anakinra (Figure S4C, Supporting Information). Moreover, we evaluated the contribution of various immune cells to FGF19‐mediated CRLM. We used the anti‐Ly‐6G neutralizing antibody, clodronate liposomes (Clod.), anti‐asialo GM1 antibody, anti‐CD20 antibody, and diphtheria toxin (DT) to deplete neutrophils, macrophages, NK cells, B cells, and DC cells, respectively. Only neutrophil depletion completely blocked FGF19‐mediated liver metastasis (Figure S4D, Supporting Information). These findings suggest that neutrophils play a critical role in the prometastatic effect of FGF19.

### 2.5 FGF19 Regulated the Formation of Metastasis‐Supporting NETs in Liver Metastatic Niches

**Figure 4:**
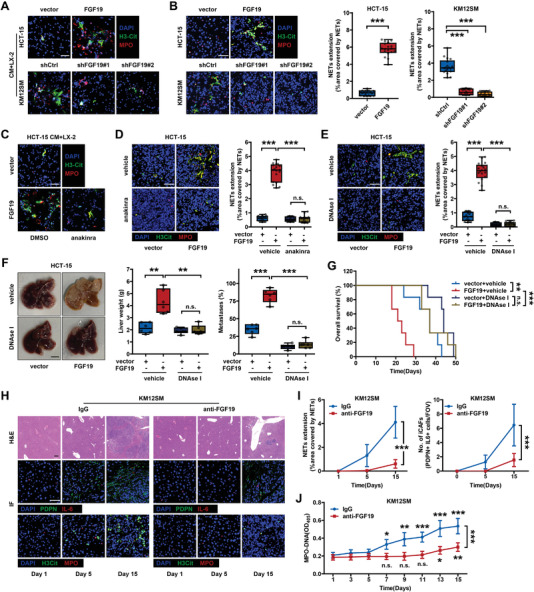
FGF19 regulated NET formation in liver metastatic niches to promote the liver metastasis of colorectal cancer. A) IF staining of NETs (H3Cit+ and MPO+ structures) formed by neutrophils treated with CM derived from FGF19‐overexpressing HCT‐15 and FGF19‐knockdown KM12SM cells for 12 h (*n* = 18 RMFs from 6 experimental replicates per group). Scale bar: 50 µm. B) IF staining and quantification of NETs in livers of mice injected with FGF19‐overexpressing HCT‐15 and FGF19‐knockdown KM12SM cells (*n* = 18 RMFs from 6 mice per group). Scale bar: 50 µm. C) IF staining of NETs formed by neutrophils cocultured with LX‐2 cells that had been pretreated with CM derived from FGF19‐overexpressing HCT‐15 cells in the presence or absence of anakinra for 12 h (*n* = 18 RMFs from 6 experimental replicates per group). Scale bar: 50 µm. D) IF staining and quantification of NETs in the livers of mice injected with FGF19‐overexpressing HCT‐15 cells with or without anakinra treatment (*n* = 18 RMFs from 6 mice per group). Scale bar: 50 µm. E–G) Liver metastasis of mice with splenic injection of FGF19‐overexpressing HCT‐15 cells, with or without DNAse I treatment (5 mg kg^−1^) (*n* = 6 mice). IF staining and quantification of NETs in the livers of mice (*n* = 18 RMFs from 6 mice per group), F) liver weight and the proportion of tumor metastases in livers, and G) animal overall survival are shown. Scale bar: 50 µm. H–J) Liver and serum specimens obtained on the indicated days after splenic injection of KM12SM cells (*n* = 6 mice). H&E staining, IF staining, and quantification of iCAFs and NETs in livers (*n* = 18 RMFs from 6 mice per group), and J) serological MPO‐DNA levels are shown. Scale bar for H&E: 200 µm. Scale bar for IF: 50 µm. *p < 0.05; **p < 0.01; ***p < 0.001; n.s., nonsignificant. In (D) and (F), data were subjected to Student's *t*‐test. In (B) and (E), data were subjected to Mann–Whitney test. In (I) and (J), data were subjected to two‐way ANOVA. In (G), data were subjected to log‐rank test. See also related Figure S5, Supporting Information.

Metastatic cancer cells can induce neutrophils to form metastasis‐supporting NETs in target organs, with liver metastases exhibiting the most abundant NETosis, which might mediate the hepatotropism of metastatic cancer cells, such as CRC and breast cancer cells.^[^ ^18^, ^40^ ^]^ To investigate whether FGF19 promotes NETosis in liver metastatic niches and determine its underlying mechanism, we first examined the induction of NETosis by FGF19 in vitro. IF staining showed that CM derived from FGF19‐overexpressing HCT‐15 cells or FGF19‐silenced KM12SM cells, respectively, enhanced or attenuated the ability of LX‐2 cells to induce NET structures, which were similar to those induced by phorbol 12‐myristate 13‐acetate (PMA) (**Figure** **4**A; Figure S5A,B, Supporting Information). Meanwhile, direct stimulation of neutrophils with FGF19‐containing cancer cell CM failed to induce NETs (Figure S5C, Supporting Information). We then detected the levels of NETs in liver metastases affected by FGF19 overexpression or knockdown in CRC cells. FGF19 overexpression in HCT‐15 cells led to increased NETs in mouse livers, whereas FGF19 knockdown in KM12SM cells had the opposite effect (Figure 4B). Moreover, anakinra was found to efficiently impede NETosis induced by FGF19‐overexpressing CRC cells in vitro and in vivo (Figure 4C,D; Figure S5D, Supporting Information). These results suggest an iCAF‐dependent regulatory mechanism of FGF19 on NETosis.

Subsequently, we assessed the promoting effect of NETs on FGF19‐induced CRLM. NET media, obtained from the supernatant of PMA‐stimulated neutrophils, increased the proliferation of HCT‐15 and KM12SM cells in vitro, which was attenuated by the degradation of NETs with DNase I or inhibition of neutrophil elastase with GW311616 (NEi) (Figure S5E,F, Supporting Information). More importantly, using an in vivo spleen‐injection liver metastasis model, we showed that digestion of NETs in mouse livers with DNase I (Figure 4E) markedly abolished FGF19‐enhanced liver metastasis (Figure 4F), thereby extending the OS of the mice (Figure 4G). These data highlight that the prometastatic function of CRC‐derived FGF19 is primarily mediated by NETosis.

### 2.6 FGF19 Promoted NET Formation in Liver Premetastatic Niches

NET formation in livers was reported to precede tumor metastasis in a mouse model. Hepatic NETs form liver‐specific premetastatic niches (PMNs) that promote liver metastasis through the capture of CTCs by NETs and the chemotaxis of CTCs by NET‐DNA.^[^ ^18^ ^]^ To investigate whether FGF19‐induced NETosis occurred prior to the colonization of metastatic CRC cells in the liver, we established an in vivo liver metastatic model with splenic injection of FGF19‐expressing KM12SM cells treated with or without FGF19 neutralizing antibody and obtained liver tissue and peripheral blood samples from mice on specific days after the injection. We found that iCAF and NET formation in mouse livers occurred as early as Day 5, which was earlier than the colonization of KM12SM cells and the increase in MPO‐DNA, a serological marker of NETs (Figure 4H–J). However, this feed‐forward mechanism was inhibited by FGF19 clearance with a neutralizing antibody (Figure 4H–J). Thus, we showed that FGF19 induces iCAF and NET formation in PMNs during CRLM.

### 2.7 FGF19 Induced NET Formation by Facilitating Complement C5a and IL‐1*β* Production in iCAFs

**Figure 5:**
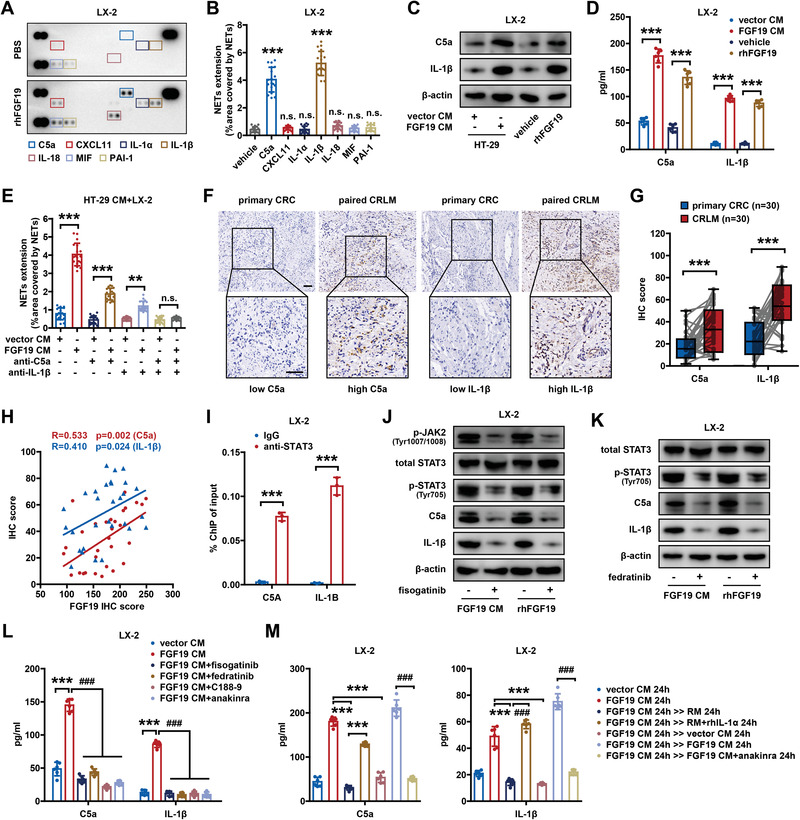
FGF19 induced NET formation by facilitating complement C5a and IL‐1*β* production in iCAFs. A) Cytokine array of the media of LX‐2 cells pretreated with rhFGF19 (50 ng mL^−1^) for 24 h. Seven factors were upregulated in the supernatant of LX‐2 cells stimulated with rhFGF19. B) Quantification of NETs formed by neutrophils stimulated with complement C5a, CXCL11, IL‐1*α*, IL‐1*β*, IL‐18, MIF, or PAI‐1 (*n* = 18 RMFs from 6 experimental replicates per group). C) Intracellular and D) extracellular expression of complement C5a and IL‐1*β* in LX‐2 cells treated with rhFGF19 or FGF19‐containing CM (*n* = 3 for WB, or 6 for ELISA). E) Quantification of NETs formed by neutrophils cocultured with LX‐2 cells that had been pretreated with FGF19‐containing CM in the presence of C5a neutralizing antibody (50 ng mL^−1^) or IL‐1*β* neutralizing antibody (1 µg mL^−1^) (*n* = 18 RMFs from 6 experimental replicates per group). F) Representative IHC staining of C5a or IL‐1*β* expression in paired primary CRC and CRLM tissues. Scale bar: 50 µm. G) Expression of complement C5a and IL‐1*β* were higher in CRLM tissues (*n* = 30) than that in paired primary CRC tissues (*n* = 30). H) Correlation between IHC scores of FGF19 and C5a or IL‐1*β* in human CRLM tissues (*n* = 30). I) ChIP‒qPCR assays showed the recruitment of STAT3 to the promoter regions of C5 and IL1B (*n* = 3). J,K) Intracellular and L) extracellular expression of C5a and IL‐1*β* in LX‐2 cells stimulated with rhFGF19 or FGF19‐containing CM and treated with or without fisogatinib (100 nм), fedratinib (10 µм), C188‐9 (5 µg mL^−1^), and anakinra (20 mg mL^−1^) (*n* = 3 for WB, or 6 for ELISA). M) Extracellular expression of complement C5a and IL‐1*β* in LX‐2 cells treated with FGF19‐containing CM for 24 h and then subjected to regular medium, regular medium with rhIL‐1*α* (1 ng mL^−1^), FGF19‐free CM, fresh FGF19‐containing CM, or anakinra for 24 h (*n* = 6). ** or ^##^ *p* < 0.01; *** or ^###^ *p* < 0.001; n.s., nonsignificant. In (B), (D), (I), (L), and (M), data were subjected to Student's *t‐*test. In (E), data were subjected to Mann–Whitney test. In (G), data were subjected to paired Student's *t*‐test. See also related Figure S6, Supporting Information.

To understand the molecular mechanism through which FGF19‐induced iCAFs regulate NETosis, we examined the secreted factors of FGF19‐conditioned LX‐2 cells using cytokine antibody arrays. Seven cytokines, namely complement C5a, CXCL11, IL‐1*α*, IL‐1*β*, IL‐18, MIF, and PAI‐1, were upregulated in the supernatant of iCAFs (**Figure** **5**A), and only C5a and IL‐1*β* could drive neutrophils to release NETs (Figure 5B). Western blotting and ELISA also validated the upregulation of C5a and IL‐1*β* in FGF19‐conditioned LX‐2 cells (Figure 5C,D; Figure S6A, Supporting Information), but not in CRC cells (Figure S6B, Supporting Information). Neutralization of C5a or IL‐1*β* partially inhibited FGF19‐induced NETosis in vitro, whereas simultaneous neutralization of both factors completely abrogated this effect (Figure 5E). Furthermore, C5a and IL‐1*β* expression was higher in liver metastases than that in the paired primary tumors (Figure 5F,G) and was positively correlated to FGF19 expression, as evidenced by IHC staining (Figure 5H).

We then investigated whether FGF19 regulated C5a and IL‐1*β* expression through the JAK2‐STAT3 signaling pathway. A chromatin immunoprecipitation (ChIP)‐ quantitative polymerase chain reaction assay was performed to confirm the interaction of STAT3 with the C5a and IL‐1*β* promoters. The levels of STAT3 bound to the C5a and IL‐1*β* promoters were statistically significant in LX‐2 cells (Figure 5I). ENCODE human STAT3 ChIP‐seq datasets also showed binding of STAT3 to the C5a and IL‐1*β* promoters (https://www.encodeproject.org). Furthermore, we examined the effect of JAK2‐STAT3 pathway inhibition on C5a and IL‐1*β* expression in FGF19‐conditioned LX‐2 cells. Inhibition of FGFR4, JAK2, and STAT3 with fisogatinib, fedratinib, and C188‐9, respectively, reduced the expression of C5a and IL‐1*β* (Figure 5J–L; Figure S6C–E, Supporting Information). More importantly, the enhancing effect of FGF19 on C5a and IL‐1*β* expression was dependent on the maintenance of the iCAF phenotype, because the disruption of iCAF polarization with anakinra reversed this effect (Figure 5M). Taken together, these findings demonstrate that FGF19 increases C5a and IL‐1*β* production in iCAFs to induce NETosis by activating the JAK2‐STAT3 signaling pathway.

### 2.8 NET Formation in Liver Metastasis Was Associated with FGF19 Expression and Liver Metastasis in Human CRC

**Figure 6:**
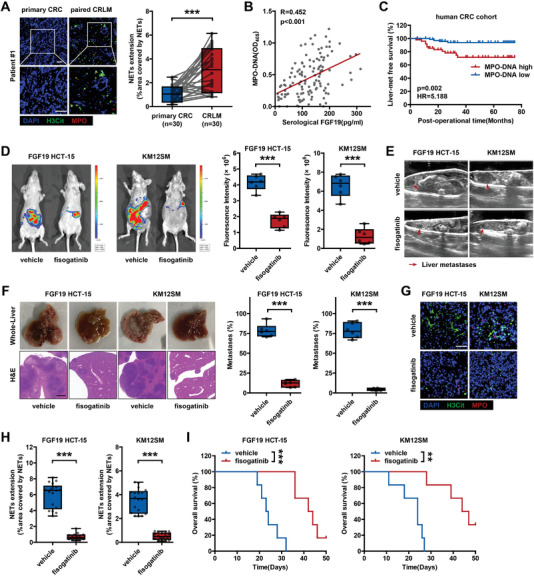
NET formation was correlated with FGF19 in liver metastases, and targeting FGF19 signaling with fisogatinib prevented CRLM. A) IF staining and quantification of NETs in primary CRC and CRLM tissues (*n* = 30). Scale bar: 50 µm. B) Correlation between serological FGF19 and MPO‐DNA (*n* = 122). C) Kaplan‒Meier survival curves of liver metastasis‐free survival of patients with CRC with low (*n* = 72) and high (*n* = 50) serological levels of MPO‐DNA. D–I) Splenic injection of KM12SM and FGF19‐overexpressing HCT‐15 cells for liver metastasis experiments (*n* = 6 mice). D) IVIS images and quantification, E) ultrasound images, F) liver weight and the proportion of tumor metastases in livers, G,H) IF staining and quantification of NETs in livers, and I) animal overall survival are shown. Scale bar for H&E: 1 mm. Scale bar for IF: 50 µm. **p < 0.01; ***p < 0.001. In (D), (F), and (H), data were subjected to Student's *t*‐test. In (A), data were subjected to paired Student's *t*‐test. In (C), data were subjected to Cox proportional hazards regression. In (I), data were subjected to log‐rank test. See also related Figure S7, Supporting Information.

We first investigated the correlation between FGF19 expression and NETosis in liver metastatic niches. We detected the levels of NETs in 30 paired pCRC and CRLM tissues via IF staining of NET markers (**Figure** **6**A). NET formation was increased in liver metastases compared with that in primary tumors and was associated with FGF19 expression (Figure 6A; Figure S7A, Supporting Information). In addition, the serological levels of MPO‐DNA were higher in patients with CRLM than that in patients with primary CRC and was positively correlated with serological FGF19 levels (Figure 6B; Figure S7B, Supporting Information). We also tested the clinical relevance of MPO‐DNA, and a high serological level was closely correlated with synchronous and subsequent liver metastasis and lower OS rates in patients with CRC (Figure 6C; Figure S7C,D, Supporting Information).

### 2.9 Targeting FGF19 Signaling with Fisogatinib Prevented CRLM in a Mouse Model

Given its crucial role and clinical significance in liver metastasis, we explored whether targeting FGF19 could serve as an efficient treatment strategy for CRLM. Fisogatinib (BLU‐554) is a highly potent and selective oral FGFR4 inhibitor. A phase I first‐in‐human trial in patients with advanced HCC expressing FGF19 validated the safety and preliminary clinical activity of fisogatinib.^[^ ^37^, ^41^ ^]^ In this study, IVIS and ultrasound imaging were used to quantify liver metastatic burden. Fisogatinib treatment of BALB/c nude mice with splenic injection of KM12SM cells and FGF19‐overexpressing HCT‐15 cells inhibited NET formation in liver metastatic niches, thereby mitigating the FGF19‐induced liver‐metastatic burden and extending animal survival (Figure 6D–I). In summary, these data confirm the role of FGF19 in regulation of the liver‐metastatic niches, and suggest that FGF19 might serve as a potential target of CRLM.

## 3 Discussion

**Figure 7:**
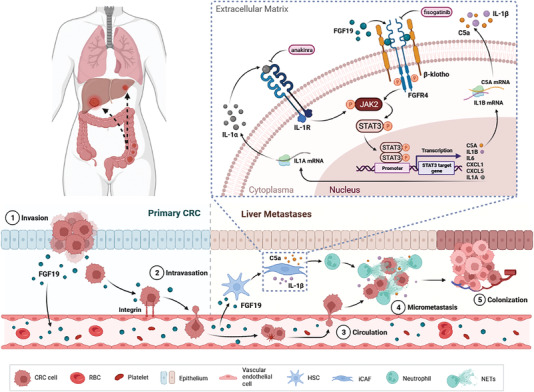
Schematic depiction of the mechanism of FGF19‐mediated colorectal cancer liver metastasis via induction of iCAF and NET formation. Created with BioRender.com.

In this study, we demonstrate the functional role of tumor‐secreted FGF19 in the liver metastasis of CRC by inducing iCAF formation and subsequent NETosis in liver metastatic niches. FGF19 activates an IL‐1*α* autocrine signal via the JAK2‐STAT3 pathway for the induction and maintenance of the iCAF phenotype. Complement C5a and IL‐1*β* secreted by FGF19‐induced iCAFs then activates neutrophils to form NETs in metastatic niches, which in turn accelerates the colonization and outgrowth of CRC cells. Additionally, our preclinical study confirmed that targeting FGF19 signaling with fisogatinib efficiently inhibits the prometastatic effect of FGF19, which provides a novel strategy for CRLM intervention (**Figure** **7**).

Liver metastasis is a complex process with numerous cellular and molecular components involved. Distinct reciprocal interactions between tumor cells and the TME are critical to liver metastasis and progression.^[^ ^42^, ^43^ ^]^ For instance, kupffer cells, the first cell population that tumor cells encounter upon entering the liver, can enhance tumor cell colonization and angiogenesis by secreting HGF and VEGF and regulate immunological tolerance by releasing immunosuppressive cytokine IL‐10.^[^ ^44^, ^45^, ^46^ ^]^ Additionally, monocytes and macrophages recruited to liver metastases promote tumor growth and angiogenesis via VEGF, EGF, FGF2, and TGF‐*β* and increase metastatic expansion through matrix metalloproteinases.^[^ ^42^ ^]^ Neutrophils are among the first myeloid cells to be recruited to metastatic niches.^[^ ^47^ ^]^ Tumor‐associated neutrophils facilitate the adhesion and outgrowth of disseminating tumor cells and suppress antitumor immunity mediated by CD8+ T cells.^[^ ^48^, ^49^, ^50^ ^]^ Therefore, liver metastasis is not mediated by a sole component; rather, the metastatic niche is probably a complex ecosystem in which the stromal and immune cells actively interact with tumor cells at every step of the metastatic cascade.

The involvement of NETs in liver metastasis has recently drawn increasing attention. NETs are abundant in the liver metastases.^[^ ^18^, ^40^ ^]^ NET‐DNA released by NETs acts as a chemokine that attracts CTCs and enhances cell motility by directly binding to the NET‐DNA receptor CCDC25.^[^ ^18^ ^]^ NETs can also induce CAF activation and promote T cell exhaustion in liver metastases, which would provide an optimizing “soil” for metastatic cell colonization.^[^ ^51^, ^52^ ^]^ Hepatic ischemia‐reperfusion injury, postoperative intra‐abdominal infection, and tumor‐secreted IL‐8 can activate neutrophils to generate NETs and accelerate the development and progression of liver metastasis.^[^ ^40^, ^53^, ^54^ ^]^ In the present study, we propose a novel regulatory mechanism for NETosis. We find that CRC‐derived FGF19 promoted the activation of neutrophils to form NET structures in a liver metastatic niche via the complement C5a and IL‐1*β* produced by iCAFs. Targeting tumor‐promoting cells in the TME is generally considered to have great clinical prospects, as normal cells have more genetic stability, which may make them easier targets compared with tumor cells with unstable genomes. However, over the past few years, the immense complexity and heterogeneity of the TME has become apparent, and these early perspectives seem to be overly optimistic, as it may be difficult to reverse an established tumor‐supporting TME by depleting only one or a few cellular components.^[^ ^55^, ^56^ ^]^ Thus, targeting the tumor‐derived molecules responsible for the induction and maintenance of the TME may be more feasible. Our study reveals that targeting FGF19 signaling with fisogatinib inhibits iCAF formation and subsequent NETosis, which disrupts the metastasis‐supportive “soil” and interferes with CRLM progression.

Overall, this study verifies the potential of FGF19 as a prognostic marker and therapeutic target. Our preclinical study showed the promising effect of targeting FGF19 signaling with fisogatinib, which is an inhibitor examined in a phase I clinical trial, for the treatment of CRLM. These data demonstrate that fisogatinib is a potential cancer therapy drug and that its use should be prioritized in the treatment of patients with CRLM exhibiting high FGF19 expression.

## 4 Experimental Section

### Patients and Specimens

Human CRC cohort consisting of 122 patients who underwent surgery in Qilu Hospital of Shandong University from 2013 to 2016 was selected following the criteria: i) patients who underwent radical resection with a clear surgical margin; ii) patients with available tumor tissues, serum samples, follow‐up information, and complete medical records; iii) patients with a postsurgical survival time of more than 1 month; and iv) patients with no history of other malignancies. The tumors were classified and staged according to the 8th AJCC/UICC TNM classification system. Overall survival (OS) was defined as the interval from initial surgical treatment to the date of death due to any cause or the date of the most recent follow‐up. Liver metastasis‐free survival (LMFS) was defined as the interval from the initial surgical treatment to the date of liver metastasis or the date of the most recent follow‐up. Informed consent was obtained from all patients. All experiments were approved and supervised by the Ethics Committee on Scientific Research of Shandong University Qilu Hospital. The information of human CRC cohort was detailed in Table S4, Supporting Information.

### Immunohistochemistry

Immunohistochemistry (IHC) staining was performed to evaluate FGF19 expression in patients’ tissues as described.^[^ ^36^, ^57^ ^]^ Paraffin‐embedded tissue sections were first deparaffinized and hydrated. EDTA (pH = 9.0) was used for antigen retrieval and 5% bovine serum albumin (BSA) was used for nonspecific antigen blocking at room temperature. Primary anti‐FGF19 antibody (1:200, ab225942, Abcam, Cambridge, UK), anti‐*β*‐klotho antibody (1:100, ab106794, Abcam), anti‐complement C5a antibody (1:500, ab281923, Abcam), and anti‐IL‐1*β* antibody (1:100, 12242, Cell Signaling Technology, Danvers, MA, USA) were applied and incubated at 4 °C overnight. Goat anti‐rabbit antibody labeled with biotin (Zsbio, Beijing, China) was used for 30 min at room temperature. The peroxidase reaction was performed with 3,3‐diaminobenzidine (DAB) solution (Solarbio, Beijing, China).

IHC score was evaluated according to previous studies.^[^ ^36^, ^57^ ^]^ The IHC score was evaluated by Quant Center software, which calculated the staining intensity and the area of each staining. IHC score = (percentage of cells of weak intensity × 1) + (percentage of cells of moderate intensity × 2) + (percentage of cells of strong intensity × 3). The cohorts were divided into subgroups with high‐ and low‐expression by the cut‐off value of IHC score, which was confirmed as the point with the highest sum of specificity and sensitivity in the receiver operating characteristic (ROC) curves.

### Cell Lines and Cell Culture

Human CRC cell lines HCT‐116, HT‐29, HCT‐15, SW480, SW620, Colo201, RKO, Caco‐2, human HSC cell line LX‐2, and murine hepatocyte cell line AML12 were purchased from The Cell Bank of Type Culture Collection of The Chinese Academy of Sciences (Shanghai, China). Human CRC cell lines KM12 and KM12SM were obtained from Meisen Chinese Tissue Culture Collections (Zhejiang, China). Cells were cultured in DMEM (Corning Life Sciences, Corning, NY, USA) medium supplemented with 10% fetal bovine serum (FBS; Gibco, Grand Island, NY, USA) and 1% penicillin‐streptomycin (Gibco) at 37 °C under 95% air and 5% CO_2_. All cell lines were authenticated using short tandem repeat (STR) analysis. Mycoplasma contamination was tested every week. Cell culture flasks/dishes/plates, round coverslips, Pasteur pipettes, and centrifuge tubes were purchased from NEST Biotechnology Co., Ltd. (Wuxi, China).

### Drugs, Recombinant Proteins, and Neutralizing Antibodies

DNase I (HY‐P72974, MedChemExpress, Monmouth Junction, NJ, USA) was used to degrade NETs and NEi (GW‐311616; HY‐15891, MedChemExpress) was used to inhibit neutrophil elastase. Fedratinib (S2736, Selleck, Houston, TX, USA) was used to inhibit JAK2, C188‐9 (S8605, Selleck) was used to inhibit STAT3, and fisogatinib (BLU‐554; S8503, Selleck) and BLU9931 (A8706, APExBIO, Houston, USA) were used to inhibit FGFR4. Clodronate liposomes (40337ES10, YEASEN Biotechnology, Shanghai, China) was used for macrophage depletion, and diphtheria toxin (D0564, Sigma‐Aldrich, St. Louis, MO, USA) was used for dendritic cell depletion.

Recombinant human FGF19, IL‐1*α*, CXCL11, MIF and PAI‐1 were purchased from R&D Systems (Minneapolis, MN, USA). Recombinant human IL‐1*β*, IL‐18, and complement C5a were purchased from Novoprotein (Shanghai, China).

Human FGF19 neutralizing antibody (AF969, R&D Systems), human IL‐1*β* neutralizing antibody (AF‐201‐NA, R&D Systems), and human complement C5a neutralizing antibody (MAB2037, R&D Systems) were used in this study. Mouse Ly‐6G antibody (16‐5931‐85, eBioscience, San Diego, CA, USA) was used for neutrophil depletion, mouse asialo GM1 antibody (16‐6507‐39, eBioscience) was used for NK cell depletion, and mouse CD20 antibody (152104, Biolegend, San Diego, CA, USA) was used for B cell depletion.

### RNA Interference

RNA knockdown was performed by transfection with small interfering RNA (siRNA; Likely Biotechnology, Beijing, China). The target sequences for siFgf15s were as follows: GCGCGGACGGCAAGATATA (siFgf15#1); GGAGGAAATGGACTGTTTA (siFgf15#2); GGAGGATGTAGACCACCTA (siFgf15#3).

5 × 10^5^ AML12 cells were seeded into 6‐well plates 6–8 h prior to transfection. siRNAs or scramble oligo (100 nм) were transfected into cells with Lipofectamine 3000 reagent (Invitrogen, Carlsbad, CA, USA), according to the manufacturer's instructions. The efficiency of knockdown was detected by WB.

### Lentivirus Vector Construction and Transfection

The full‐length sequence or shRNA of FGF19 were constructed into the lentivirus vector (GeneChem, Shanghai, China). The target sequences for shFGF19s were as follows: ACTTGTCTGATCATAACATTG (shFGF19#1); CCTGGGACAACTTGAGAATTC (shFGF19#2).

Transfection experiments were executed according to the manufacturer's instructions. Briefly, 5 × 10^5^ CRC cells were seeded into 6‐well plates the day before transfection. The lentivirus was premixed with 40 µL HiTransG P (GeneChem) and subsequently added to CRC cells with 1 mL complete DMEM medium. After 24 h, the medium was replaced with fresh culture medium. Stable cell lines were selected using puromycin (8 µg mL^−1^) incubation for 7 days. The efficiency of knockdown and overexpression were verified by WB.

### In Vivo Experiment

All animal experiments were approved by the Institutional Animal Care and Use Committee (IACUC) of Cancer Hospital, Chinese Academy of Medical Sciences (NCC2022A377). 6‐week‐old female BALB/c mice were purchased from Gempharmatech Laboratory (Nanjing, China). Spleen injection was performed following the previously published procedures.^[^ ^58^ ^]^ Briefly, after anesthesia, the spleen of mouse was exteriorized by a laparotomy. For the spleen injection, 2.5 × 10^6^ HT‐29 or KM12SM cells suspended in 50 µL PBS were injected into the distal tip of spleens with an insulin syringe (BD, Franklin Lakes, NJ, USA). A whitening bulge of the spleen should be observed upon injection area. After the whitening bulge disappeared, the spleen was repositioned and the abdominal wound was then sutured. Animals were kept until death or until the end of this experiment (50 days). A live imaging system (IVIS Spectrum; PerkinElmer) was used to detect the in vivo liver metastases. An in vivo ultrasound imaging was performed to evaluate the echogenicity of mouse liver as described.^[^ ^59^ ^]^ The major parameters as follows: ML6‐15 linear transducer; center frequency of 15.0 MHz; MI of 0.6; dynamic range of 63 dB; focus of 0.75 cm; depth of 2.5 cm. Liver metastases were counted and confirmed by H&E staining.

AAV‐Tbg‐shFgf15 and AAV‐shCtrl were purchased from Likely Biotechnology (Beijing, China). The viruses (1.0 × 10^12^ genome copies per mouse) were injected into BALB/c mice via the tail vein. Four weeks after virus injection, the mice were subjected to splenic injection of control or FGF19‐overexpressing HCT15 cells. The efficiency of knockdown and overexpression were verified by WB.

For rhFGF19 treatment, mice were i.p. injected with rhFGF19 proteins (50 µg kg^−1^, R&D Systems) daily. For FGF19 blockade, mice were i.p. injected with a goat polyclonal FGF19‐neutralizing antibody (25 µg per mouse, AF969, R&D Systems) every other day. For anakinra treatment, mice were i.p. injected with anakinra (500 mg per mouse, 250 mg morning and 250 mg evening, Kineret) 3 days prior to spleen injection, followed by daily injection until the termination of experiment. For fisogatinib treatment, mice were given fisogatinib (BLU‐554; 10 mg kg^−1^, S8503, Selleck) orally daily.

For neutrophil depletion, mice were i.p. injected with Ly‐6G antibody (200 µg per mouse, 16‐5931‐85, eBioscience) 48 hours prior to spleen injection, followed by injection of the reagents every 3 days until the termination of experiment. For macrophage depletion, mice were i.v. injected with clodronate liposomes (200 µL per mouse, 40337ES10, YEASEN Biotechnology) 24 h prior to spleen injection, followed by injection of the reagents every 4 days until the termination of experiment. For NK cell depletion, mice were i.p. injected with asialo GM1 antibody (50 µg per mouse, 16‐6507‐39, eBioscience) 24 h prior to spleen injection, followed by injection of the reagents every 5 days until the termination of experiment. For B cell depletion, mice were i.p. injected with CD20 antibody (250 µg per mouse, 152104, Biolegend) 48 h prior to spleen injection, followed by weekly injection until the termination of experiment. For DC cell depletion, mice were i.p. injected with diphtheria toxin (20 ng g^−1^, D0564, Sigma‐Aldrich) 48 h prior to spleen injection, followed by injection of the reagents with dose of 4 ng g^−1^ every other day. For DNase I treatment, mice were i.p. injected with DNase I (5 mg kg^−1^, HY‐P72974, MedChemExpress) 24 h prior to spleen injection, followed by daily injection of the reagents until the termination of experiment.

### Western Blotting

Western blotting (WB) was performed as described.^[^ ^36^, ^57^ ^]^ Cells with a density of about 70–80% could be used for protein extraction. Total protein was extracted using RIPA lysis buffer containing 1% PMSF and 1% phosphatase inhibitor (Beyotime, Shanghai, China). After centrifugation at 12 000 rpm at 4 °C, the supernatant was collected for protein concentration detection using a bicinchoninic Acid (BCA) Assay Kit (Beyotime). After denaturation, the protein extract was resolved by 10% SDS‐PAGE and transferred to PVDF membrane (0.22 µm, Millipore, Bedford, MA, USA). PVDF membrane was incubated with diluted primary antibody at 4 °C overnight, and then incubated with the secondary antibody at room temperature for 2 h. After adding chemiluminescent solution (Millipore), the membrane was evaluated on a chemifluorescence detection system (Amersham Imager 600, GE). Western blotting bands were quantitatively analyzed by Image J (NIH). SDS‐PAGE gel fast preparation kit and primary antibody dilution buffer were purchased from Shandong Sparkjade Biotechnology Co., Ltd.

Primary antibodies and dilution ratios were listed in Table S5, Supporting Information.

### Quantitative Real‐Time PCR

Quantitative real‐time PCR (qRT‐PCR) was performed as described.^[^ ^36^, ^57^ ^]^ All reactions were done in a 20 µL reaction volume in triplicate. TRIzol reagent (Thermo Fisher Scientific, Waltham, MA, California, USA) was used to extract total RNA from cells or tissues samples. A reverse transcriptase kit (TOYOBO, Osaka, Japan) was used for cDNA synthesis and SYBR Green Master Mix (Roche, Mannheim, Germany) was used for real‐time PCR. Ct values were calculated by a Light Cycler Roche 480 PCR instrument. The 2^−ΔΔCt^ method was introduced for the comparison between groups, and GAPDH was used as internal control. Primers used for qPCR are listed in Table S6, Supporting Information.

### Enzyme‐Linked Immunosorbent Assay

Enzyme‐linked immunosorbent assay (ELISA) kits were used to detect the concentrations of human FGF19 (DF1900, R&D Systems), IL‐1*α* (DLA50, R&D Systems), IL‐1*β* (DLB50, R&D Systems) and complement C5a (ab193695, Abcam) in culture supernatants or serum as described.^[^ ^36^ ^]^ The absorbance values could be read on a microplate reader (Molecular Devices) at a wavelength of 450 nm, within 30 min. The standard curve was used to convert absorbance values to protein concentrations.

### CCK8, Colony Formation, Wound Healing and Transwell Assays

CCK8, colony formation, wound healing and transwell assays of HCT‐15, HT‐29, and KM12SM cells were performed as described.^[^ ^57^ ^]^

### Conditioned Medium

The generation of conditioned medium (CM) was performed as described.^[^ ^36^ ^]^ Briefly, HCT‐15, KM12SM or LX‐2 cells were cultured in DMEM complete medium until the cell density was 50–60%. Then the complete medium was replaced with serum‐free medium and the cells were cultured for 48 h. The supernatant of culture medium was collected and centrifuged for 1000 g to discard the pellets. The supernatant was 10× concentrated with Amicon Ultra‐15 mL filters (10 kDa, Millipore) at 4000 g. The CM could be used for the subsequent in vitro HSCs polarization and NET formation assays.

### In Vitro Hepatic Stellate Cell Polarization

Human hepatic stellate cell line LX‐2 was cultured in DMEM complete medium at 37 °C under 95% air and 5% CO_2_. For hepatic stellate cell polarization in vitro, LX‐2 cells were treated with CRC‐derived CM, rhFGF19 proteins (50 ng mL^−1^, R&D Systems) or rhIL‐1*α* (1 ng mL^−1^, R&D Systems) for 24 h. Human FGF19 neutralizing antibody (10 µg mL^−1^, AF969, R&D Systems), FGFR4 inhibitor fisogatinib (100 nм, S8503, Selleck) and BLU9931 (10 µм, A8706, APExBIO), JAK2 inhibitor fedratinib (10 µм, S2736, Selleck), STAT3 inhibitor C188‐9 (5 µg mL^−1^, S8605, Selleck), and anakinra (20 mg mL^−1^, Kineret) were used to inhibit iCAFs formation. WB, qRT‐PCR and ELISA were performed to detect the expression of myCAFs markers (*α*‐SMA, ACTG2, COL1A1, and COL2A1) and iCAFs markers (IL1A, IL1B, IL6, CXCL1, CXCL5), and the phosphorylation of JAK2‐STAT3 signaling pathway. Flow cytometry (FC) was performed to detect the expression of iCAF surface marker PDGFR*α* (1:250, 3174, Cell Signaling Technology) and myCAF marker *α*‐SMA (1:200, ab7817, Abcam). Data was collected and analyzed on a FACSCalibur flow cytometer (BD) FlowJo software (USA). Immunofluorescence (IF) staining was performed to detect the expression of pan‐CAF marker podoplanin (PDPN; 1:500, ab10288, Abcam) and iCAF marker IL‐6 (1:100, ab233706, Abcam). Images were acquired on a fluorescence microscope (Olympus).

### Neutrophil Isolation

Neutrophil isolation was performed following the previously published procedures.^[^ ^18^ ^]^ Briefly, human neutrophils were isolated from the peripheral blood of healthy donors using Ficoll density gradient centrifugation. Under sterile conditions, neutrophils were resuspended in DMEM supplemented with 10% FBS and cultured in 24‐well plate in the density of 2.5 × 10^5^ mL^−1^, at 37 °C under 95% air and 5% CO_2_.

### Separate Co‐Culture System

For the separate co‐cultured system, 1 × 10^5^ LX‐2 cells were seeded into the 24‐well chamber insert (8 µm, Corning) and pretreated with CRC‐derived CM, while 2.5 × 10^5^ neutrophils were seeded in the 24‐well plate. After separate culture for 24 h, the insert with LX‐2 cells was put into 24‐well plate containing neutrophils for indicated period to establish a iCAFs‐neutrophils separate co‐culture system.

### Two‐Chamber Neutrophil Migration Assay

Neutrophil migration assay was performed using 24‐well plate with 8.0 µm‐pore transwell chamber inserts (Corning). 2.5×10^5^ neutrophils were seeded into the 24‐well plate and treated with medium from LX‐2 cells cultured in CRC cell CM. After 36 hours incubation, cells attached to the bottom of the chambers were fixed with paraformaldehyde for 20 min at 4 °C and then stained with 0.5% crystal violet (Beyotime) for 30 minutes at room temperature. Images of five random visual fields of microscopy at 200× magnification were exported to Image J (NIH) for cell counting.

### In Vitro NET Formation Assay

For NET formation assays in vitro, 2.5 × 10^5^ neutrophils in 24‐well plate were incubated with CRC cell‐derived CM, recombinant protein (IL‐1*α*, IL‐1*β*, IL‐18, complement C5a, MIF and PAI‐1), PMA (500 nм, Beyotime) or co‐cultured with CAFs through a CAFs‐neutrophils separate co‐culture system. After 12 h of incubation or co‐culture, neutrophils were fixed with 4% paraformaldehyde at 4 °C for 15 min and the nonspecific antigen was blocked with 5% goat serum at 37 °C for 1 h. Primary goat anti‐MPO (1:50, AF3667, R&D Systems) and rabbit anti‐H3Cit (1:200, ab5103, Abcam) antibodies were applied and incubated at 4 °C overnight. CY3‐conjugated donkey anti‐goat (1:200, Servicebio) and FITC‐conjugated donkey anti‐rabbit (1:200, Servicebio) secondary antibodies were used for 2 h at room temperature. After mounting, NETs could be observed with the fluorescence microscope (Olympus). The percentage of the field of view positive for the green signal (H3Cit) was regarded as the level of NETs formation and was measured using Image J (NIH), as previously described.

### Immunofluorescence of Paraffin‐Embedded Tissues

Immunofluorescence (IF) staining was performed as described.^[^ ^60^ ^]^ After antigen retrieval and antigen blocking, the sections were incubated with diluted primary antibodies at 4 °C overnight. Then the sections were incubated with CY3‐ or FITC‐conjugated secondary antibodies (1:2000, Thermo Fisher Scientific) at room temperature for 2 h. Finally, the sections were mounted in anti‐fade mounting medium with DAPI (Beyotime). The levels of NETs were evaluated as above.

Primary antibodies and dilution ratios were listed in Table S7, Supporting Information.

### Detection of Serological MPO‐DNA

Serological MPO‐DNA was detected using a capture ELISA method as previously described.^[^ ^61^ ^]^ 96‐well microtiter plates were coated with anti‐MPO monoclonal antibody (5 µg mL^−1^, 0400‐0002, Bio‐Rad) as the capturing antibody overnight at 4 °C. After blocking with 1% BSA, serum was added into the wells together with peroxidase‐labelled anti‐DNA monoclonal antibody (component no. 2 of the Cell Death Detection ELISA kit, 11774425001, Roche). After incubation for 2 h at room temperature, the peroxidase substrate (11774425001, Roche) was added. The absorbance values were measured at 405 nm using the microplate reader.

### Chromatin Immunoprecipitation

Chromatin immunoprecipitation (ChIP) was conducted using an enzymatic ChIP kit (9003, Cell Signaling Technology) according to the manufacturer's instructions. Briefly, 4 × 10^6^ LX‐2 cells were fixed by 1% formaldehyde for 10 min at room temperature to crosslink the protein and DNA. Micrococcal Nuclease was added and incubated for 20 min at 37 °C with frequent mixing for DNA digestion. The lysate was centrifuged to collect the chromatin pellets. The chromatin samples were incubated with anti‐phospho‐STAT3‐Tyr705 (1:50, 9145, Cell Signaling Technology) antibody at 4 °C overnight. Rabbit IgG (2729, Cell Signaling Technology) was used as negative control. A non‐immunoprecipitated sample (2%) was used as input control. The purified DNA was then detected by qPCR. The primers used for ChIP‐qPCR were listed in Table S8, Supporting Information.

### Cytokine Antibody Array

Cytokines were detected in media of LX‐2 cells stimulated with rhFGF19 proteins by using the Proteome Profiler Human Cytokine Array Kit (ARY005B, R&D Systems) according to the manufacturer's instruction. Briefly, LX‐2 cells were stimulated with rhFGF19 (50 ng mL^−1^, R&D Systems) for 24 h. The culture supernatants were collected, centrifuged for 1000 × *g* to discard the pellets and 10× concentrated with Amicon Ultra‐15 mL filters (10 kDa, Millipore) at 4000 × *g*. Array membranes, previously spotted with capture antibodies by the manufacturer, were incubated with 0.5 mL of culture supernatants overnight at 4 °C. After washing three times, the membranes were incubated with Streptavidin‐HRP for 30 min, and revealed using Chemi‐Reagent Mix. The protein signal was detected with a chemifluorescence detection system (Amersham Imager 600, GE). Quantitative analysis of specific protein expression was performed using Image J.

### RNA Isolation and mRNA‐Sequencing

Total messenger RNA (mRNA) of LX‐2 cells with or without rhFGF19 stimulation were isolated using the TRIzol reagent (Thermo Fisher Scientific). After undergoing quality control, 1 unstimulated and 2 rhFGF19‐stimutaed (50 ng mL^−1^ or 100 ng mL^−1^) cell samples were sequenced by Illumina HiSeq 4000 (LC‐Bio Technologies Co., Ltd., Hangzhou, China). The sequence reads were mapped to reference genome using HISAT2 software. Raw read counts were converted to log_2_counts‐per‐million (logCPM) to ensure that the data followed a normal distribution. Differentially expressed genes (DEGs) were selected based on the criteria as a log2 |fold change|>1. The heatmap was created by the pheatmap R package. GO (Gene Oncology) analysis was performed by the clusterProfiler R package and visualized by the ggplot2 R package. GSEA (Gene Set Enrichment Analysis) was performed by the GSEA software (version 4.3.0).

Raw data are available at the GEO database (GSE215882). List of DEGs is in Table S3, Supporting Information.

### Bioinformation Analysis

The Human Genome Microarray datasets from the previous study were downloaded from the GEO database (GEO: GSE87211, GSE14095, GSE41568). Differential expression analysis of mRNAs was conducted using DEseq2 of R package. Venn diagram was used to overlap DEGs from different datasets.

### Statistical Analysis

Data were presented as mean ± standard deviation (SD) of no smaller than three biological replicates. SPSS Statistics 23 and GraphPad Prism 5.0 software were used for statistical analysis and chart generation. Differences between two independent groups were assessed using Student's *t*‐test when normally distributed (determined by D'Agostino‐Pearson tests), including Welch correction in case of unequal variances (ascertained using *F*‐test). If not normal, a nonparametric test (Mann–Whitney test) was used. Two‐way ANOVA was performed to analyze the difference of multiple groups. The survival curves were plotted using the Kaplan‐Meier method, and the Cox‐proportional hazards regression was used to determine the effect of biomarkers on OS (overall survival) and LMFS (liver metastasis‐free survival). *p*‐values < 0.05 were considered statistically significant.

## Conflict of Interest

The authors declare no conflict of interest.

## Author Contributions

C.L. and T.C. contributed equally to this work. C.L. and T.C. contributed equally to this work. C.L., T.C., J.L., and Y.W. carried out our research. X.W. and J.L. designed all of the experiments. C.Z., L.G., D.S., and T.Z. collected the specimens and perform the follow‐up. T.C. and X.W. participated in data analysis and interpretation. C.L., T.C., and J.L. wrote the manuscript. All of the authors have seen and commented on the manuscript.

## Supporting information

Supporting Information

Supplemental Table 1

Supplemental Table 2

Supplemental Table 3

## Data Availability

The data that support the findings of this study are openly available in GEO at reference number GSE215882.
