# Supplementary material for: FGF19‐Induced Inflammatory CAF Promoted Neutrophil Extracellular Trap Formation in the Liver Metastasis of Colorectal Cancer
Source: Adv Sci (Weinh). 2023 Jun 22;10(24):2302613. doi: 10.1002/advs.202302613 (PMC10460854; doi:10.1002/advs.202302613)
Supplement: Supplementary file 1 — Supporting Information [file ADVS-10-2302613-s004.pdf]

ADVANCED SCIENCE
Open Access

Supporting Information

for *Adv. Sci.*, DOI 10.1002/advs.202302613

FGF19-Induced Inflammatory CAF Promoted Neutrophil Extracellular Trap Formation in the Liver Metastasis of Colorectal Cancer

Chen Li, Tianli Chen, Jialiang Liu, Yue Wang, Chunhuan Zhang, Lu Guo, Dandan Shi, Tingguo Zhang, Xishan Wang and Jie Li**

Supporting Information

FGF19-induced inflammatory CAF promoted neutrophil extracellular trap formation in the liver metastasis of colorectal cancer

Chen Li et al.

Figure S1. Related to Figure 1.

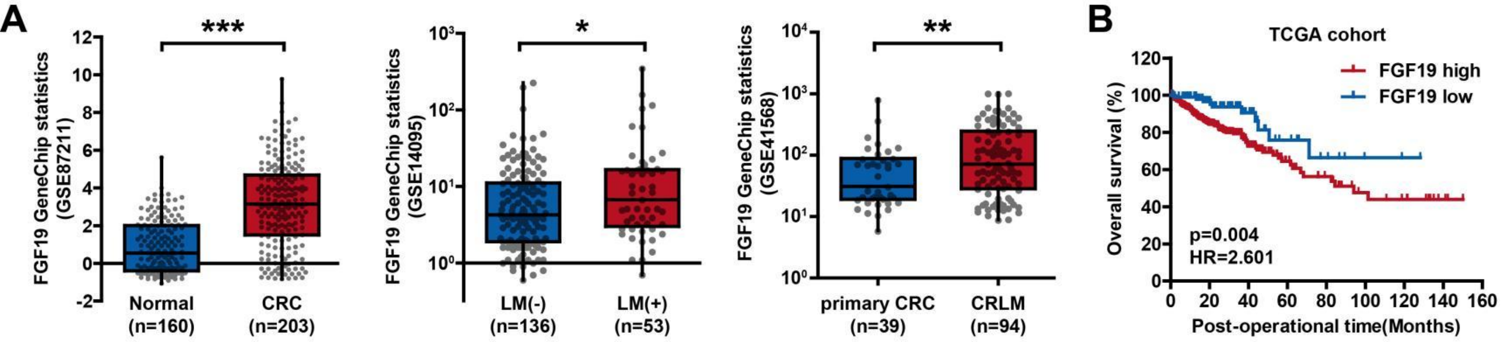

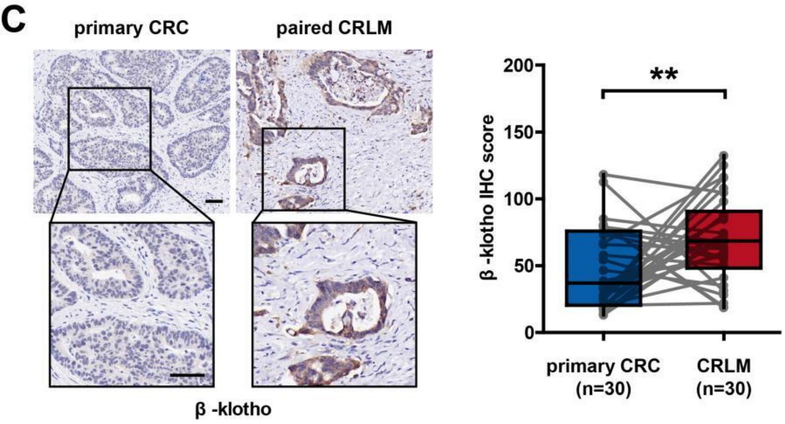

D

Correlations between FGF19 expression and liver metastasis.

	CRC	FGF19 Low	FGF19 High	p value
**Synchronous liver metastasis**				
(+)	110	57	53	
(-)	12	2	10	0.021*
**Subsequent liver metastasis**				
(+)	106	57	49	
(-)	16	2	14	0.002*

* Calculated with Chi-square test.

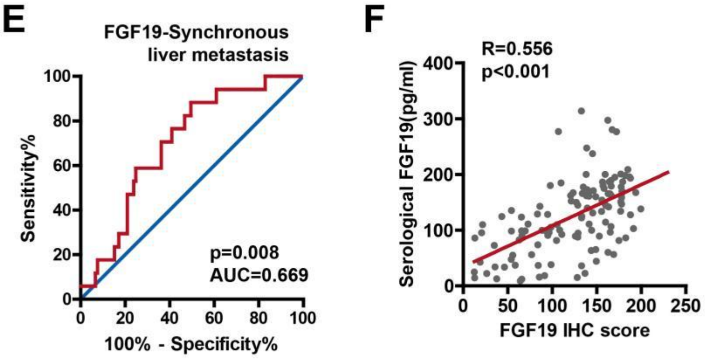

G

Correlations between serological FGF19 and liver metastasis.

	CRC	sFGF19 Low	sFGF19 High	p value
**Synchronous liver metastasis**				
(+)	110	68	42	
(-)	12	3	9	0.014*
**Subsequent liver metastasis**				
(+)	106	67	39	
(-)	16	4	12	0.004*

* Calculated with Chi-square test.

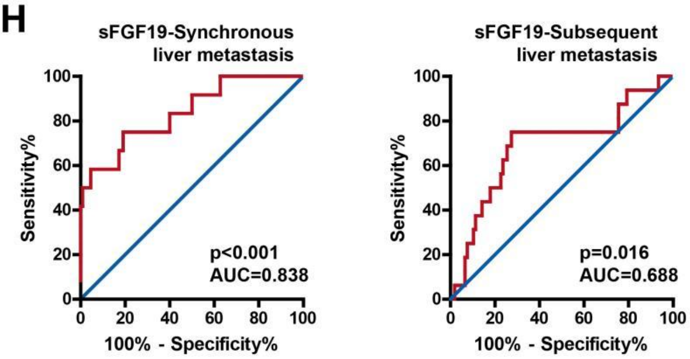

Figure S1. Related to Figure 1.

(A) The expression of FGF19 was higher in CRC tissues than in normal tissues (GSE87211), higher in CRC tissues with liver metastasis (LM(+)) than in CRC tissues without liver metastasis (LM(-)), and higher in CRLM than in primary CRC tissues (GSE41568).

(B) Kaplan-Meier survival curves of overall survival for CRC patients with low (*n*=247) and high (*n*=161) FGF19 expression. Patients were stratified to FGF19-high or low groups by the cutoff value.

(C) Representative IHC staining of β-klotho expression in paired primary CRC and CRLM tissues. β-klotho expression was higher in CRLM tissues (*n*=30) than that in primary CRC tissues (*n*=30). Scale bar: 50 μm.

(D) The correlation between FGF19 expression and liver metastasis was analyzed with Chi-square test.

(E) Receiver operator characteristic (ROC) curve to predict liver metastasis from FGF19 expression in CRC tumors. AUC: area under curve.

(F) Correlation between the histological and serological FGF19 (*n*=122).

(G) The correlation between serological FGF19 levels and liver metastasis was analyzed with Chi-square test.

(H) ROC curve to predict liver metastasis from serological FGF19 levels.

* $p<0.05$; ** $p<0.01$; *** $p<0.001$. In (A), data were subjected to Mann-Whitney test. In (C), data were subjected to paired Student's *t*-test.

Figure S2. Related to Figure 2.

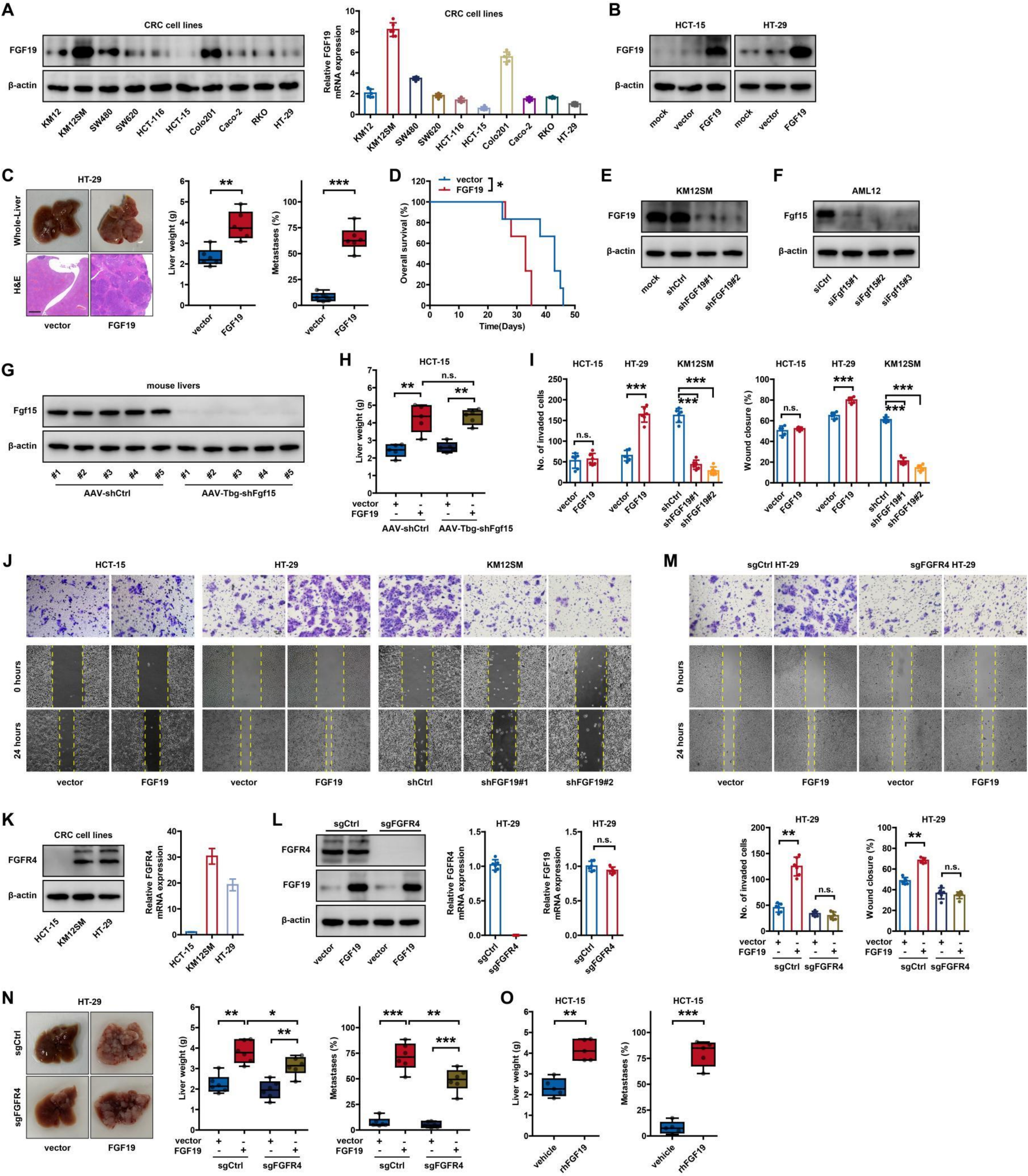

Figure S2. Related to Figure 2.

(A) The expression of FGF19 in different CRC cell lines was detected with WB (n=3) and qPCR (*n*=6).

(B) FGF19 was overexpressed in HCT-15 and HT-29 cells with lentivirus encoding full-length FGF19.

(C and D) Spleen injection of HT-29 cells with FGF19 overexpression for liver metastasis experiments (*n*=6 mice). Liver weight and the proportion of tumor metastases in livers **(C)**, and animal overall survival **(D)** are shown. Scale bar: 1 mm.

(E) FGF19 was knocked down in KM12SM cells with two different shRNAs.

(F) Fgf15 was knocked down in murine AML12 cells with three different siRNAs.

(G) Fgf15 was knocked down in mouse livers with AAV-Tbg-shFgf15 (*n*=5 mice).

(H) Mice were administrated intravenously with AAV-shCtrl or AAV-Tbg-shFgf15 for 4 weeks and then injected FGF19-overexpressing HCT-15 cells for liver metastasis experiments (*n*=5 mice). Liver weight is shown.

(I and J) Migration and invasion of HCT-15, HT-29 and KM12SM cells were detected with wound healing and transwell assays after regulating FGF19 expression (*n*=6).

(K) The expression of FGFR4 in HCT-15, HT-29 and KM12SM cells was detected with WB (*n*=3) and qPCR (*n*=6).

(L) FGF19 was knocked out in HT-29 cells by CRISPR/Cas9 with a single guide RNA (sgRNA) (*n*=3 for WB, or 6 for qRT-PCR).

(M) Migration and invasion of FGFR4-knocking out HT-29 cells were detected with wound healing and transwell assays after FGF19 overexpression (*n*=6).

(N) Spleen injection of FGFR4 knocking-out HT-29 cells with FGF19 overexpression for liver metastasis experiments (*n*=6 mice). Liver weight and the proportion of tumor metastases in livers were shown. Scale bar: 1 mm.

(O) Mice were pretreated with rhFGF19 and sequentially splenic-injected with HCT-15 cells. Liver weight and the proportion of tumor metastases in livers are shown (*n*=5 mice).

* $p<0.05$; ** $p<0.01$; *** $p<0.001$. n.s., nonsignificant. In (C), (H), (I), (L) (M), (N),

and (O), data were calculated by Student's *t*-test.

Figure S3. Related to Figure 3.

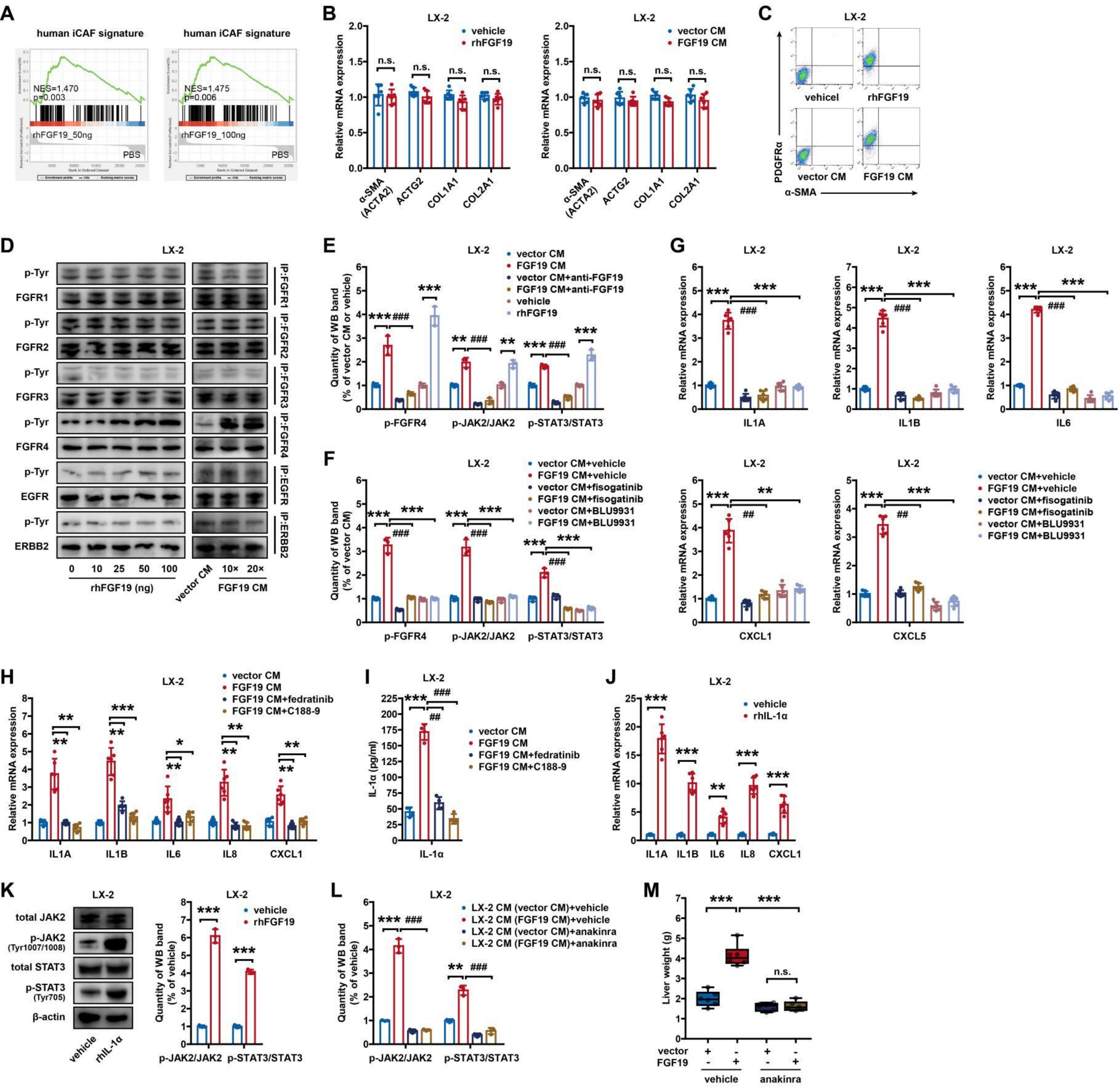

Figure S3. Related to Figure 3.

(A) GSEA plot of iCAF signature using mRNA-seq data of LX-2 cells. NES: normalized enrichment score.

(B) mRNA expression of myCAF markers in LX-2 cells treated with rhFGF19 or FGF19-containing CM for 24 hours (*n*=6).

(C) Flow cytometry analysis of PDGFRα and α-SMA expression in LX-2 cells treated with rhFGF19 or FGF19-containing CM for 24 hours.

(D) LX-2 cells were incubated with FGF19-containing CM or rhFGF19, and immunoprecipitated with FGFR1-4, EGFR, and ERBB2 antibody. The output receptors and pan-phosphorylated-Tyr levels were detected with WB.

(E) The quantity of WB bands in Figure 3H (*n*=3).

(F) The quantity of WB bands in Figure 3I (*n*=3).

(G) mRNA expression of iCAF markers in LX-2 cells treated with FGF19-containing CM in the presence or absence of fisogatinib or BLU9931 (*n*=6).

(H) mRNA expression of iCAF markers in LX-2 cells treated with FGF19-containing CM, in the presence or absence of fedratinib and C188-9, for 24 hours (*n*=6).

(I) Extracellular expression of IL-1α in LX-2 cells stimulated with FGF19-containing CM, and treated with or without fedratinib and C188-9 (*n*=4).

(J) mRNA expression of iCAF markers in LX-2 cells treated with rhIL-1α for 24 hours (*n*=6).

(K) Phosphorylation of JAK2 and STAT3 in LX-2 cells treated with rhIL-1α for 24 hours (*n*=3).

(L) The quantity of WB bands in Figure 3L (*n*=3).

(M) Quantification of liver weight is shown (*n*=6 mice), related to Figure 3P.

* or $^{\#}$ $p<0.05$; ** or $^{\#\#}$ $p<0.01$; *** or $^{\#\#\#}$ $p<0.001$. n.s., nonsignificant. In (B) and (E-M), data were calculated by Student's *t*-test.

Figure S4.

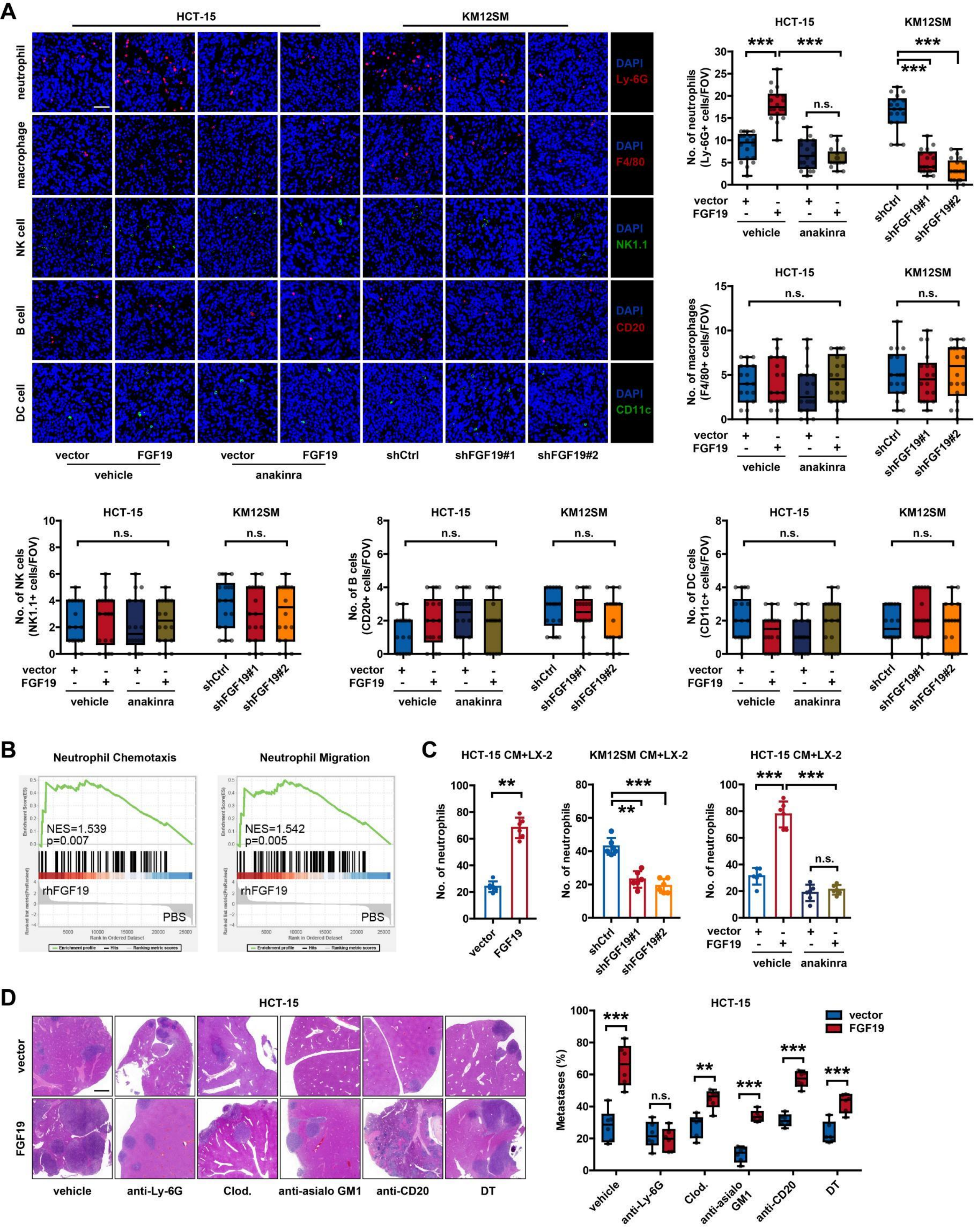

A
HCT-15
KM12SM
neutrophil
macrophage
NK cell
B cell
DC cell
DAPI
Ly-6G
F4/80
NK1.1
CD20
CD11c
vector
FGF19
vehicle
anakinra
shCtrl
shFGF19#1
shFGF19#2
No. of neutrophils (Ly-6G+ cells/FOV)
No. of macrophages (F4/80+ cells/FOV)
No. of NK cells (NK1.1+ cells/FOV)
No. of B cells (CD20+ cells/FOV)
No. of DC cells (CD11c+ cells/FOV)
***
n.s.
B
Neutrophil Chemotaxis
Neutrophil Migration
NES=1.539
p=0.007
NES=1.542
p=0.005
rhFGF19
PBS
C
HCT-15 CM+LX-2
KM12SM CM+LX-2
No. of neutrophils
**
D
HCT-15
vector
FGF19
vehicle
anti-Ly-6G
Clod.
anti-asialo GM1
anti-CD20
DT
Metastases (%)

Figure S4.

(A) IF staining of Ly-6G+ neutrophils, F4/80+ macrophages, NK1.1+ NK cells, CD20+ B cells and CD11c+ DC cells in the livers of mice injected with FGF19-overexpressing HCT-15 cells with or without anakinra, or FGF19-knocking down KM12SM cells (*n*=18 RMFs from 6 mice per group). Scale bar: 50 μm.

(B) GSEA plots showed that neutrophil-related signatures were enriched in rhFGF19-stimulated LX-2 cells.

(C) Migration of human neutrophils attracted by LX-2 cells pretreated with CM derived from FGF19-overexpressing HCT-15 or FGF19-knocking down KM12SM cells for 12 hours (*n*=6).

(D) Liver metastasis of mice with spleen injection of FGF19-overexpressing HCT-15 cells, with or without the treatment of anti-Ly-6G antibody, clodronate liposomes (Clod.), anti-asialo GM1 antibody, anti-CD20 antibody and diphtheria toxin (DT) (*n*=6 mice). Representative H&E staining and the proportion of tumor metastases are shown. Scale bar: 1 mm.

** $p<0.01$; *** $p<0.001$. n.s., nonsignificant. In (A), (C) and (D), data were calculated by Student's t test.

Figure S5. Related to Figure 4.

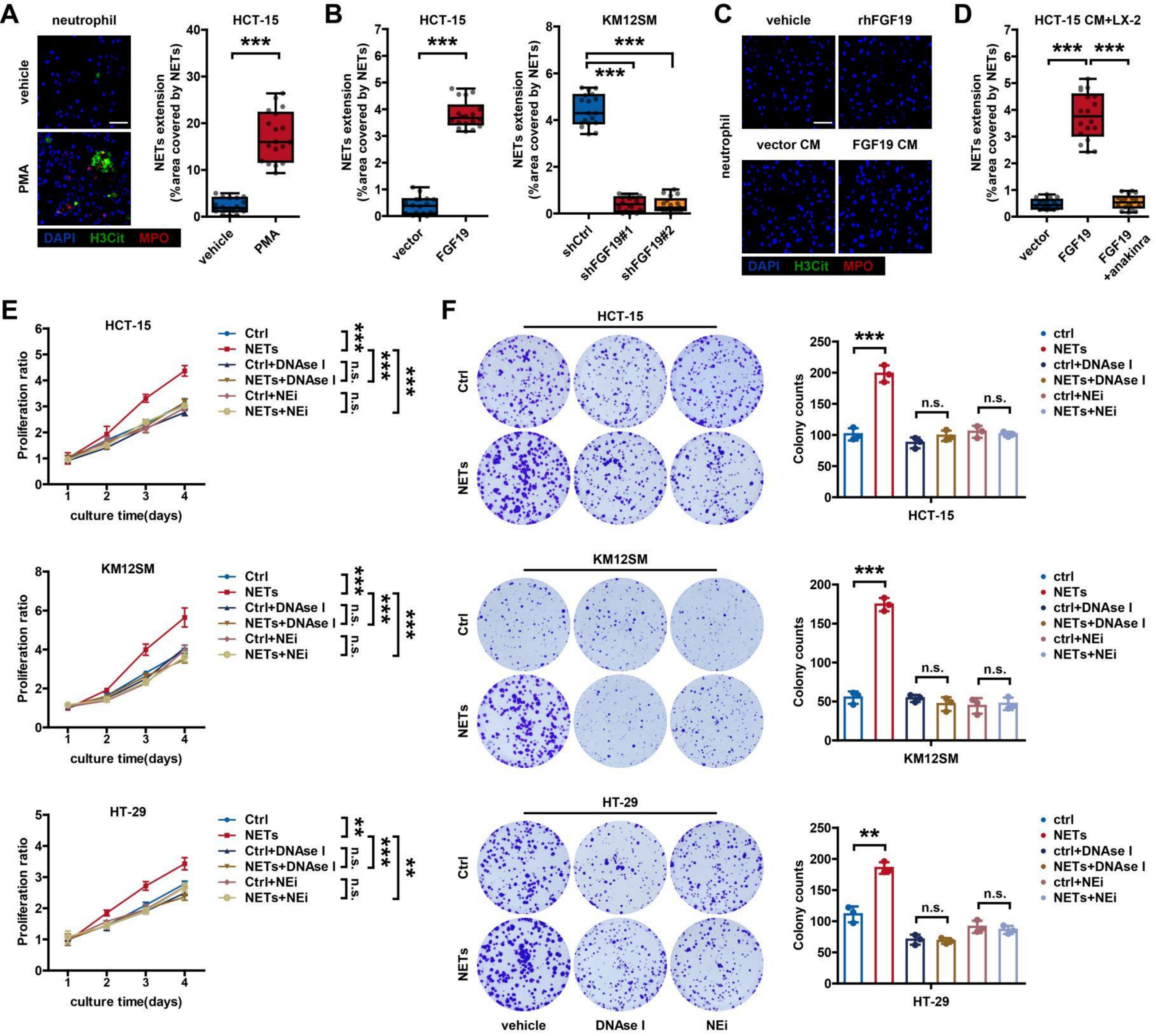

Figure S5. Related to Figure 4.

(A) Representative images of IF staining and quantification of NET structures stimulated by PMA (*n*=18 RMFs from 6 experimental replicates per group).

(B) Quantification of NETs (H3Cit+ and MPO+ structures) formed by neutrophils treated with CM derived from FGF19-overexpressing HCT-15 and FGF19-knockdown KM12SM cells for 12 hours (*n*=18 RMFs from 6 experimental replicates per group), related to Figure 4A.

(C) IF staining of neutrophils treated directly with rhFGF19 or CM derived from FGF19-overexpressing HCT-15 cells for 12 hours. Scale bar: 50 μm.

(D) Quantification of NETs formed by neutrophils cocultured with LX-2 cells that had been pretreated with CM derived from FGF19-overexpressing HCT-15 cells in the presence or absence of anakinra for 12 hours (*n*=18 RMFs from 6 experimental replicates per group), related to Figure 4C.

(E and F) CCK8 (E) and colony formation (F) assays showed that PMA-stimulated NET media increased cell proliferation in HCT-15 and KM12SM cells, and this effect was abolished by DNase I or NEi (*n*=3).

*** p<0.001. n.s., nonsignificant. In (A), (B), (D) and (F), data were calculated by Student's *t*-test. In (E), data were calculated by two-way ANOVA.

Figure S6. Related to Figure 5.

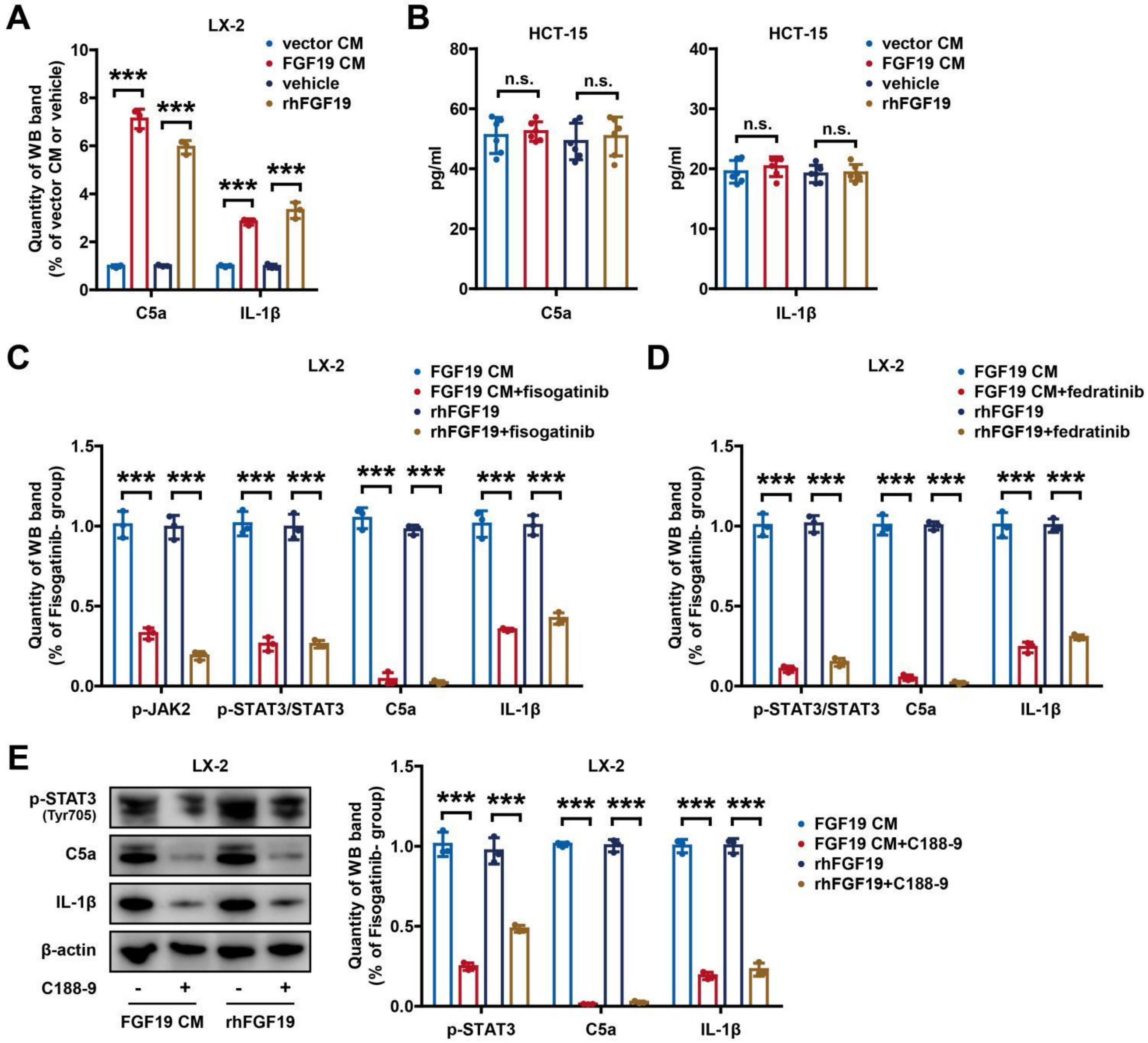

Figure S6. Related to Figure 5.

(A) The quantity of WB bands in Figure 5C (*n*=3).

(B) Extracellular expression of complement C5a and IL-1β in HCT-15 cells with FGF19 overexpression or rhFGF19 stimulation (*n*=6).

(C and D) The quantity of WB bands in Figure 5J and Figure 5K (*n*=3).

(E) Intracellular expression of complement C5a and IL-1β in LX-2 cells stimulated with rhFGF19 or FGF19-containing CM and treated with or without C188-9 (*n*=3).

***, $p<0.001$. n.s., nonsignificant. In (A), (B), (C), (D) and (E), data were calculated by Student's *t*-test.

Figure S7. Related to Figure 6.

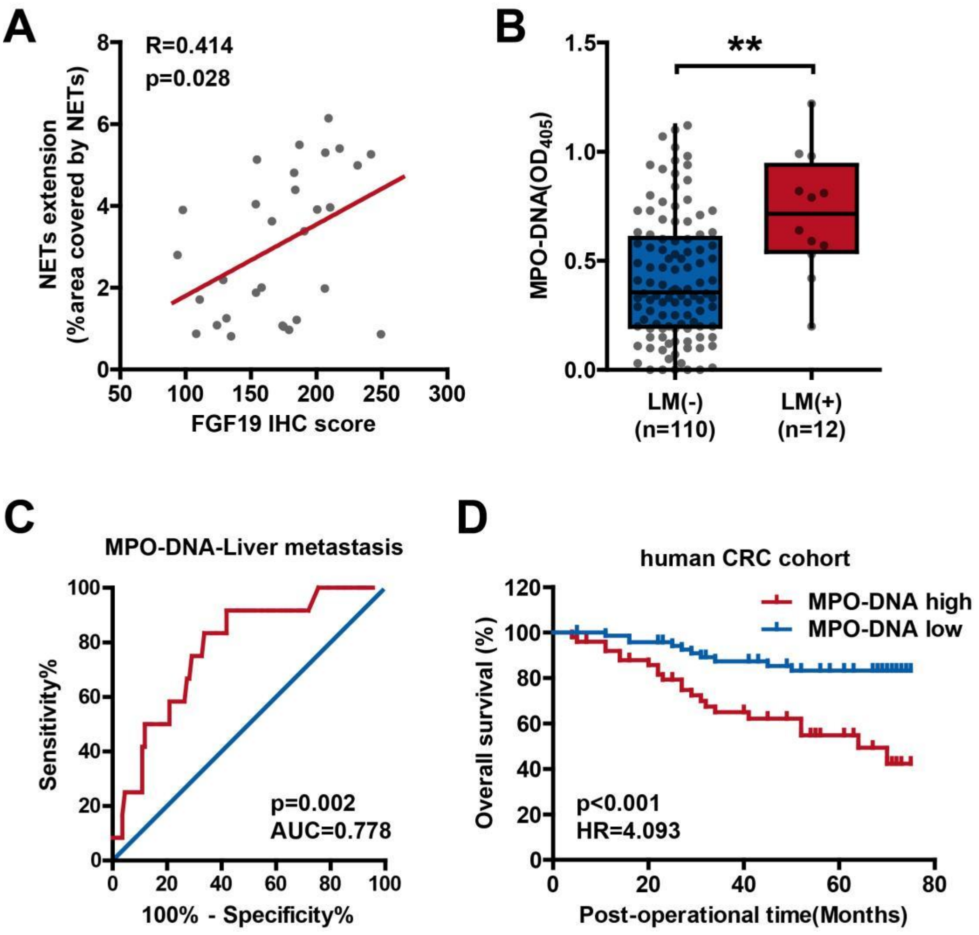

Figure S7. Related to Figure 6.

(A) Correlation between FGF19 expression and NETs levels in liver metastases (*n*=30).

(B) Serological level of MPO-DNA was higher in LM(+) CRC patients (*n*=12) than in LM(-) CRC patients (*n*=110).

(C) ROC curve to predict liver metastasis from serological MPO-DNA levels.

(D) Kaplan-Meier survival curves of overall survival for CRC patients with low (n=72) and high (n=50) serological level of MPO-DNA.

** represented $p<0.01$. In (B), data were calculated by Mann-Whitney test. In (D), data were calculated by Cox proportional hazards regression.

Table S1. FGFR4 expression in human colorectal cancer cell lines. Data was obtained from the DepMap portal (https://depmap.org/portal/).

Detailed in Excel file.

Table S2. FGFR4 expression in human colorectal cancer cell lines. Data was obtained from the GEO database (GEO ID: GSE59857).

Detailed in Excel file.

Table S3. Results of mRNA sequencing on LX-2 cells with the treatment of 50 or 100ng rhFGF19 proteins (GEO ID: GSE215882).

Detailed in Excel file.

Table S4. Information of TCGA cohort and human CRC cohort.

Clinicopathologic parameters		TCGA cohort		human CRC cohort	
		n	Percentage	n	Percentage
Age (years)	<65	146	35.78%	64	52.46%
	≥65	262	64.22%	58	47.54%
Sex	Male	213	52.20%	81	66.39%
	Female	195	47.80%	41	33.61%
Differentiation	Well			25	20.49%
	Moderately			71	58.20%
	Poorly			26	21.31%
T stage	T1-2	79	19.36%	15	12.30%
	T3-4	329	80.64%	107	87.70%
N stage	N0	244	59.80%	77	63.11%
	N1-2	164	40.20%	45	36.89%
M stage	M0	343	84.07%	105	86.07%
	M1	65	15.93%	17	13.93%
TNM stage	I-II	235	57.60%	66	54.10%
	III-IV	173	42.40%	56	45.90%
FGF19	Low	247	60.54%	59	48.36%
	High	161	39.46%	63	51.64%
Serological FGF19	Low			71	58.20%
	High			51	41.80%

Table S5. Primary antibodies for Western blotting.

Antibodies	Source	Identifier	Dilution
**FGF19**	Abcam	ab225942	1:500
**phospho-FGFR4-Tyr642**	Signalway Antibody	11836	1:1000
**JAK2**	Cell Signaling Technology	3230	1:1000
**phospho-JAK2-Tyr1007/1008**	Cell Signaling Technology	3771	1:1000
**STAT3**	Abcam	ab68153	1:2000
**phospho-STAT3-Tyr705**	Cell Signaling Technology	9145	1:1000
**complement C5a**	Abcam	ab281923	1:1000
**IL-1β**	Abcam	ab9722	1:1000
**FGFR1**	Cell Signaling Technology	9740	1:1000
**FGFR2**	Abcam	ab10648	1:2000
**FGFR3**	Cell Signaling Technology	4574	1:1000
**FGFR4**	Cell Signaling Technology	8562	1:1000
**EGFR**	Cell Signaling Technology	4267	1:1000
**ERBB2**	Cell Signaling Technology	2165	1:1000
**phospho-Tyrosine**	Santa Cruz Biotechnology	sc-7020	1:1000
**β-actin**	Cell Signaling Technology	4970	1:4000

Table S6. Primer sequences of qRT-PCR.

Genes	Forward primer(5'-3')	Reverse primer(5'-3')
**FGF19**	CTGGAGATCAAGGCAGTCGC	TGCTTCTCGGATCGGTACAC
**IL1A**	CTTCTGGGAAACTCACGGCA	AGCACACCCAGTAGTCTTGC
**IL1B**	CAACAAGTGGTGTTCTCCATGTC	ACACGCAGGACAGGTACAGA
**IL6**	CAATGAGGAGACTTGCCTGGT	GCAGGAACTGGATCAGGACT
**CXCL1**	TTGCCTCAATCCTGCATCCC	GTTGGATTTGTCACTGTTCAGCAT
**CXCL5**	GACCACGCAAGGAGTTCATC	GGAGGCTACCACTTCCACCT
**ACTA2**	CCTGACTGAGCGTGGCTATT	GCCCATCAGGCAACTCGTAA
**ACTG2**	GCAGGCTTCGCAGGAGATGATG	TGGTGATGATGCCGTGTTCAATGG
**COL1A1**	TAAAGGGTCACCGTGGCTTC	GGGAGACCGTTGAGTCCATC
**COL2A1**	GGAGCAGCAAGAGCAAGGAGAAG	TGGACAGCAGGCGTAGGAAGG
**GAPDH**	GCACCGTCAAGGCTGAGAAC	TGGTGAAGACGCCAGTGGA

Table S7. Primary antibodies for IF.

Antibodies	Source	Identifier	Dilution
MPO	R&D Systems	AF3667	1:50
H3Cit	Abcam	ab5103	1:200
human PDPN	Abcam	ab10288	1:100
mouse PDPN	Abcam	ab256559	1:500
human IL-6	Abcam	ab23306	1:100
mouse IL-6	Abcam	ab208113	1:100
Ly-6G	eBioscience	16-5931-85	1:200
F4/80	Cell Signaling Technology	30325	1:200
NK1.1	Cell Signaling Technology	39197	1:100
CD20	Cell Signaling Technology	98708	1:100
CD11c	Cell Signaling Technology	97585	1:100

Table S8. Primers for ChIP assays.

Gene	Primer	Sequence（5′ to 3′）
**C5a**	Forward	CCCGAATCCATCCCAAGCC
	Reverse	CTTCTAGGCGGTGAGCGTC
**IL1B**	Forward	GTGGACATCAACTGCACAACGAT
	Reverse	GCTTCTTAGGGGAGGGGACT
